# Association of Short-term Air Pollution Exposure With SARS-CoV-2 Infection Among Young Adults in Sweden

**DOI:** 10.1001/jamanetworkopen.2022.8109

**Published:** 2022-04-20

**Authors:** Zhebin Yu, Tom Bellander, Anna Bergström, Joakim Dillner, Kristina Eneroth, Magnuz Engardt, Antonios Georgelis, Inger Kull, Petter Ljungman, Göran Pershagen, Massimo Stafoggia, Erik Melén, Olena Gruzieva, and the BAMSE COVID-19 Study Group

**Affiliations:** 1 Institute of Environmental Medicine, Karolinska Institutet, Stockholm, Sweden; 2 Centre for Occupational and Environmental Medicine, Region Stockholm, Stockholm, Sweden; 3 Medical Diagnostics Karolinska, Karolinska University Hospital, Stockholm, Sweden; 4 SLB-analys, Environment and Health Administration, Stockholm, Sweden; 5 Department of Clinical Sciences and Education, Karolinska Institutet, Södersjukhuset, Stockholm, Sweden; 6 Department of Pediatrics, Sachs Children’s Hospital, Stockholm, Sweden; 7 Department of Cardiology, Danderyd Hospital, Stockholm, Sweden; 8 Department of Epidemiology, Lazio Regional Health Service, Rome, Italy

## Abstract

**Importance:**

Mounting ecological evidence shows an association between short-term air pollution exposure and COVID-19, yet no study has examined this association on an individual level.

**Objective:**

To estimate the association between short-term exposure to ambient air pollution and SARS-CoV-2 infection among Swedish young adults.

**Design, Setting, and Participants:**

This time-stratified case-crossover study linked the prospective BAMSE (Children, Allergy Milieu, Stockholm, Epidemiology [in Swedish]) birth cohort to the Swedish national infectious disease registry to identify cases with positive results for SARS-CoV-2 polymerase chain reaction (PCR) testing from May 5, 2020, to March 31, 2021. Case day was defined as the date of the PCR test, whereas the dates with the same day of the week within the same calendar month and year were selected as control days. Data analysis was conducted from September 1 to December 31, 2021.

**Exposures:**

Daily air pollutant levels (particulate matter with diameter ≤2.5 μm [PM_2.5_], particulate matter with diameter ≤10 μm [PM_10_], black carbon [BC], and nitrogen oxides [NOx]) at residential addresses were estimated using dispersion models with high spatiotemporal resolution.

**Main Outcomes and Measures:**

Confirmed SARS-CoV-2 infection among participants within the BAMSE cohort. Distributed-lag models combined with conditional logistic regression models were used to estimate the association.

**Results:**

A total of 425 cases were identified, of whom 229 (53.9%) were women, and the median age was 25.6 (IQR, 24.9-26.3) years. The median exposure level for PM_2.5_ was 4.4 [IQR, 2.6-6.8] μg/m^3^ on case days; for PM_10_, 7.7 [IQR, 4.6-11.3] μg/m^3^ on case days; for BC, 0.3 [IQR, 0.2-0.5] μg/m^3^ on case days; and for NOx, 8.2 [5.6-14.1] μg/m^3^ on case days. Median exposure levels on control days were 3.8 [IQR, 2.4-5.9] μg/m^3^ for PM_2.5_, 6.6 [IQR, 4.5-10.4] μg/m^3^ for PM_10_, 0.2 [IQR, 0.2-0.4] μg/m^3^ for BC, and 7.7 [IQR, 5.3-12.8] μg/m^3^ for NOx. Each IQR increase in short-term exposure to PM_2.5_ on lag 2 was associated with a relative increase in positive results of SARS-CoV-2 PCR testing of 6.8% (95% CI, 2.1%-11.8%); exposure to PM_10_ on lag 2, 6.9% (95% CI, 2.0%-12.1%); and exposure to BC on lag 1, 5.8% (95% CI, 0.3%-11.6%). These findings were not associated with NOx, nor were they modified by sex, smoking, or having asthma, overweight, or self-reported COVID-19 respiratory symptoms.

**Conclusions and Relevance:**

The findings of this case-crossover study of Swedish young adults suggest that short-term exposure to particulate matter and BC was associated with increased risk of positive PRC test results for SARS-CoV-2, supporting the broad public health benefits of reducing ambient air pollution levels.

## Key Points

**Question:**

Is exposure to residential short-term air pollution associated with SARS-CoV-2 infection in young adults?

**Findings:**

In this case-crossover study of 425 participants with SARS-CoV-2 infection identified within a Swedish population-based birth cohort, short-term air pollution exposure was associated with increased risk of SARS-CoV-2 infection despite relatively low levels of air pollution exposure.

**Meaning:**

These findings suggest that air pollution may play a role in COVID-19 and support the potential benefit of reducing air pollutant levels.

## Teaser

This case-crossover study examines the association between short-term exposure to air pollution estimated at the individual residence level and the risk of SARS-CoV-2 infection among Swedish young adults.

## Introduction

As of February 2022, the COVID-19 pandemic has resulted in more than 410 million confirmed cases and has caused more than 5.8 million deaths globally.^1^ Concerns have been raised about whether ambient air pollution could increase the risk of infection with SARS-CoV-2 as well as the severity of disease after infection because air pollution has long been recognized as a potential contributor to respiratory infectious diseases such as influenza,^2^ severe acute respiratory syndrome,^3^ and dengue.^4^

Two key pathways for the plausible mechanism between air pollution and COVID-19–related outcomes have been summarized^5^: (1) modifying host susceptibility to infection and/or disease severity and (2) elevating the risk of comorbidities. The former pathway can be mediated through upregulation of proteins critical to viral entry^6,7^ and by immune system suppression due to oxidative stress,^8^ epithelial damage,^9^ and pulmonary inflammation.^10^ Complementary to the experimental evidence, emerging ecological studies linking short-term (daily variation) exposure to air pollution and aggregated population-level data suggest a relevant role of air pollution in SARS-CoV-2 infection^11,12^; however, previous short-term studies lack individual-level exposure data, termed *ecological fallacy*.^13,14^ Statistically, the correlation tends to be overestimated when the association is assessed at the group level rather than at the individual level.^15^ A recent study^16^ indicated that ecological analyses are prone to showing spurious associations between air pollution and COVID-19. Furthermore, little is known regarding the association between short-term exposure to air pollution and COVID-19 among young adults.^12^ No single study or subgroup analysis has been reported among this age group yet, although young adults have been considered the major spreader of the virus since autumn 2020.^17^

The purpose of this study was to examine the association between short-term exposure to air pollution estimated at the individual residence level and the risk of SARS-CoV-2 infection among Swedish young adults. In addition, we assessed whether this association differs by sex, having overweight, having asthma, smoking status, season, and self-reported COVID-19–related respiratory symptoms.

## Method

### Study Population and Outcome

This case-crossover study was based on the BAMSE (Children, Allergy Milieu, Stockholm, Epidemiology [in Swedish]) population-based birth cohort including 4089 newborns in 1994 to 1996. Details on study design, recruitment procedures, and data collection have been provided elsewhere.^18^ Follow-ups were conducted at the ages of 1, 2, 4, 8, 12, 16, and 24 years. From 2016 to 2019, the 24-year follow-up^19^ was conducted with a total of 2270 participants both attending the clinical examination and questionnaire survey. Starting August 1, 2020, these participants were further invited to a new COVID-19 follow-up,^18,20^ which includes a web questionnaire from August to November 2020 (phase 1), a clinical examination and a new web questionnaire from October 2020 to June 2021 (phase 2), and an ongoing web questionnaire (phase 3). In the present study, cases with confirmed positive results for SARS-CoV-2 polymerase chain reaction (PCR) testing (for the presence of the virus’s genetic material or its fragments to detect active infection) to March 31, 2021, within the BAMSE cohort were identified through data linkage of unique personal identifier codes to the SmiNet,^21^ which is the national registry of infectious diseases in Sweden.^22^ Clinical data and self-reported COVID-19–related respiratory symptoms were obtained from questionnaire data.^20^ This study was approved by the Swedish Ethical Review Authority, and all participants gave written informed consent. This study adhered to the Strengthening the Reporting of Observational Studies in Epidemiology (STROBE) reporting guideline with a completed checklist for observational studies in epidemiology.

### Air Pollution Exposure Assessment

Daily ambient air pollutant levels (particulate matter with diameter ≤10 μm [PM_10_]; particulate matter with diameter ≤2.5 μm [PM_2.5_]; black carbon [BC]; and nitrogen oxides [NOx]) at the individual residential address were calculated using a Gaussian air quality dispersion model and a wind model, both part of the Airviro air quality management system,^23^ described elsewhere.^24,25^ Briefly, an emission inventory including local emissions from road traffic (exhaust and nonexhaust), residential wood combustion, energy production, industrial processes, and other sources (eg, off-road machinery and agriculture) in Stockholm and Uppsala counties was used as input to the dispersion modeling. In this region, road traffic is the dominant source of air pollutants. Road traffic exhaust emissions were described with emission factors for different vehicle and road types according to the European emission model HBEFA (Handbook Emission Factors for Road Transport), version 4.1.^26^ Emissions of wear particles were calculated using the NORTRIP model,^27,28^ which accounts for the number of vehicles with studded winter tires, sanding and salting of the road surface, and precipitation. In Stockholm during the late winter and in connection with dry road surfaces, the contribution from studded tire wear can be 80% to 90% of the total PM_10_ levels. Meteorological data for the wind model were taken from a 50-m mast in Högdalen in southern Stockholm and a 24-m mast outside Uppsala. The geographical distributions of air pollution levels were calculated with a Gaussian model at 2 m above open ground on a fixed grid of 250 × 250 m. In addition, a street canyon contribution was added for addresses in street segments with more than 3000 vehicles per day and multistory houses on one or both sides using the OSPM (operational street pollution model) street canyon model.^29^ To ensure that the modeled concentrations also include air pollution transported into the modeling domain from sources outside the Stockholm-Uppsala region, we added the daily mean concentrations of the respective species collected at strategically located rural monitoring sites outside Stockholm and Uppsala. Comparison of the calculated levels with measurements of daily mean values at a traffic monitoring site and 2 urban background sites (1 for BC) resulted in *R*^2^ values of 0.90 for PM_10_, 0.97 for PM_2.5_, 0.90 for BC, and 0.91 for NOx. We subsequently assigned the daily air pollution exposure preceding the case and control dates as long as 7 days (lag 0 to lag 7) to the cases as the main exposure.

### Covariates

Demographic and COVID-19–related characteristics were derived from the questionnaire data of the BAMSE 24-year follow-up and the BAMSE COVID-19 follow-up. Demographic information including age, sex, educational level (university or elementary and/or high school), occupation (student, employed, or other), current smoking (yes or no), body mass index category (having overweight or not [calculated as weight in kilograms divided by height in meters squared], where overweight was defined as ≥25) were derived from the BAMSE 24-year follow-up (before the pandemic). Asthma is defined based on at least 2 of the 3 following criteria: (1) symptoms of wheezing in the last 12 months before the date of questionnaire follow-up; (2) ever having a physician’s diagnosis of asthma; and (3) asthma medicine used occasionally or regularly in the last 12 months. This definition has been developed by a panel of experts within the Mechanisms of the Development of Allergy consortium.^30^ In the present study, individuals fulfilling the asthma definition at any follow-up to 24 years were classified as having asthma.

### Statistical Analysis

Data were analyzed from September 1 to December 31, 2021. We used a case-crossover study design widely used for analyzing short-term exposures for acute events,^31^ where each case serves as their own control at different periods, hence controlling for time-invariant (or slowly varying over time) individual confounding factors. We adopted the time-stratified strategy for control selection in which, for each case, 3 to 4 control days were selected as the same day of the week within the same calendar month and year as the case day (the day of the PCR test for each individual with SARS-CoV-2 infection). We fitted conditional logistic regression models to estimate the association between air pollution and the risk of SARS-CoV-2 infection. Air pollution exposure was modeled using linear terms in the main analysis. We also applied natural splines to investigate the exposure-response shapes. To estimate lagged associations, we applied distributed-lag models^32^ from lag 0 (day of the PCR test or control day) to lag 7 (7 days before PCR test or control) and imposed a quadratic polynomial constraint using the formula Log[E(Y)] = Covariate + λ Stratum + η_0_W_0_ + … + η_d_W_d_, where stratum is the time stratum in the time-stratified case-crossover design; W_d_ was defined as weighted sums of the air pollution exposure variable and its lags, with W_d_ = Z_1_ + 2^d^Z_2_ +…q^d^Z_q_ and W_0_ = Z_0_ + Z_1_ + …Z_q_; and the coefficient of Ws will be the parameters of the polynomial distributed lags.

Separate single-pollutant models were established for PM_2.5_, PM_10_, BC, and NOx. We also repeated the analysis using cumulative air pollution exposures. Meteorological factors such as temperature and humidity were not adjusted because they were used as in-data in the daily air pollution modeling. Further, we examined interaction by sex, having overweight, asthma, smoking status, season, and self-reported COVID-19–related respiratory symptoms by adding exposure-modifier interaction terms into the model. In sensitivity analysis, we increased the maximum lag to 14 days. Relative risks (RRs) with corresponding 95% CIs were presented for an IQR increase in air pollution concentrations. All analyses were performed using R software, version 4.0.5 (R Foundation for Statistical Computing), with 2-sided *P* < .05 indicating statistical significance.

## Results

A total of 425 participants with positive SARS-CoV-2 PCR test results were identified within the BAMSE cohort from May 5, 2020, to March 31, 2021. The descriptive statistics of the background characteristics of included participants are presented in Table 1. The median age was 25.6 (IQR, 24.9-26.3) years; 229 (53.9%) were women and 196 (46.1%) were men. Comparison between the included samples and the BAMSE original cohort as well as the 24-year follow-up are presented in eTable 1 in Supplement 1.

**Table 1.** Characteristics of Participants With Positive Results of Polymerase Chain Reaction Testing for SARS-CoV-2

Characteristic	Participant data (N = 425)^a^
Sex	
Women	229/425 (53.9)
Men	196/425 (46.1)
Age, median (IQR), y	25.6 (24.9-26.3)
Educational level attained	
University	115/345 (33.3)
Elementary or high school	230/345 (66.7)
Occupation	
Student	164/345 (47.5)
Employed	154/345 (44.6)
Other	27/345 (7.8)
Current smoking	79/345 (22.9)
Having overweight	61/278 (21.9)
Having asthma	126/408 (30.9)
COVID-19–related characteristic	
Any symptoms (any of the below)	107/200 (53.5)
Fever	76/144 (52.8)
Cough	81/145 (55.9)
Sore throat	78/144 (54.2)
Loss of taste or smell	70/146 (47.9)
Runny nose	115/146 (78.8)
Nasal congestion	96/146 (65.7)
Breathing difficulties	37/145 (25.5)
COVID-19 cases in household	81/142 (57.0)
Regularly meeting people during pandemic	147/200 (73.5)
Use of public transportation during pandemic	62/200 (31.0)

^a^ Unless otherwise indicated, data are expressed as number/total number (%) of participants. The total number is smaller for some variables owing to missing data.

The distribution of daily air pollution exposure is provided in Table 2, with a slightly higher median concentrations on the case days compared with those on control days (for PM_2.5_, 4.4 [IQR, 2.6-6.8] μg/m^3^ vs 3.8 [IQR, 2.4-5.9] μg/m^3^; for PM_10_, 7.7 [IQR, 4.6-11.3] μg/m^3^ vs 6.6 [IQR, 4.5-10.4] μg/m^3^; for BC, 0.3 [IQR, 0.2-0.5] μg/m^3^ vs 0.2 [IQR, 0.2-0.4] μg/m^3^; and for NOx, 8.2 [5.6-14.1] μg/m^3^ vs 7.7 [IQR, 5.3-12.8] μg/m^3^). Temporal variations of air pollution as well as the temperature during the study period are shown in eFigure 1 in Supplement 1. Concurrent estimates of PM_2.5_, PM_10_, and BC were highly correlated with each other (Spearman correlation index, 0.8-0.9) and moderately correlated with NOx (Spearman correlation index, 0.2-0.3) (eFigure 2 in Supplement 1).

**Table 2.** Distribution of Daily Air Pollution Exposure Levels on Case Days and Control Days

Exposure	Mean (range), μg/m^3^	Median (IQR) [difference], μg/m^3^
Case days		
PM_10_	8.8 (1.1-53.7)	7.7 (4.6-11.3) [6.7]
PM_2.5_	5.0 (0.8-23.8)	4.4 (2.6-6.8) [4.2]
BC	0.3 (0.1-1.5)	0.3 (0.2-0.5) [0.3]
NOx	11.5 (1.5-65.6)	8.2 (5.6-14.1) [8.5]
Control days		
PM_10_	8.4 (1.3-54.6)	6.6 (4.5-10.4) [5.9]
PM_2.5_	4.6 (0.7-26.1)	3.8 (2.4-5.9) [3.5]
BC	0.3 (0.03-1.5)	0.2 (0.2-0.4) [0.2]
NOx	11.0 (1.4-98.7)	7.7 (5.3-12.8) [7.5]

Abbreviations: BC, black carbon; NOx, nitrogen oxides; PM_2.5_, particulate matter with diameter ≤2.5 μm; PM_10_, particulate matter with diameter ≤10 μm.

**Figure 1.**
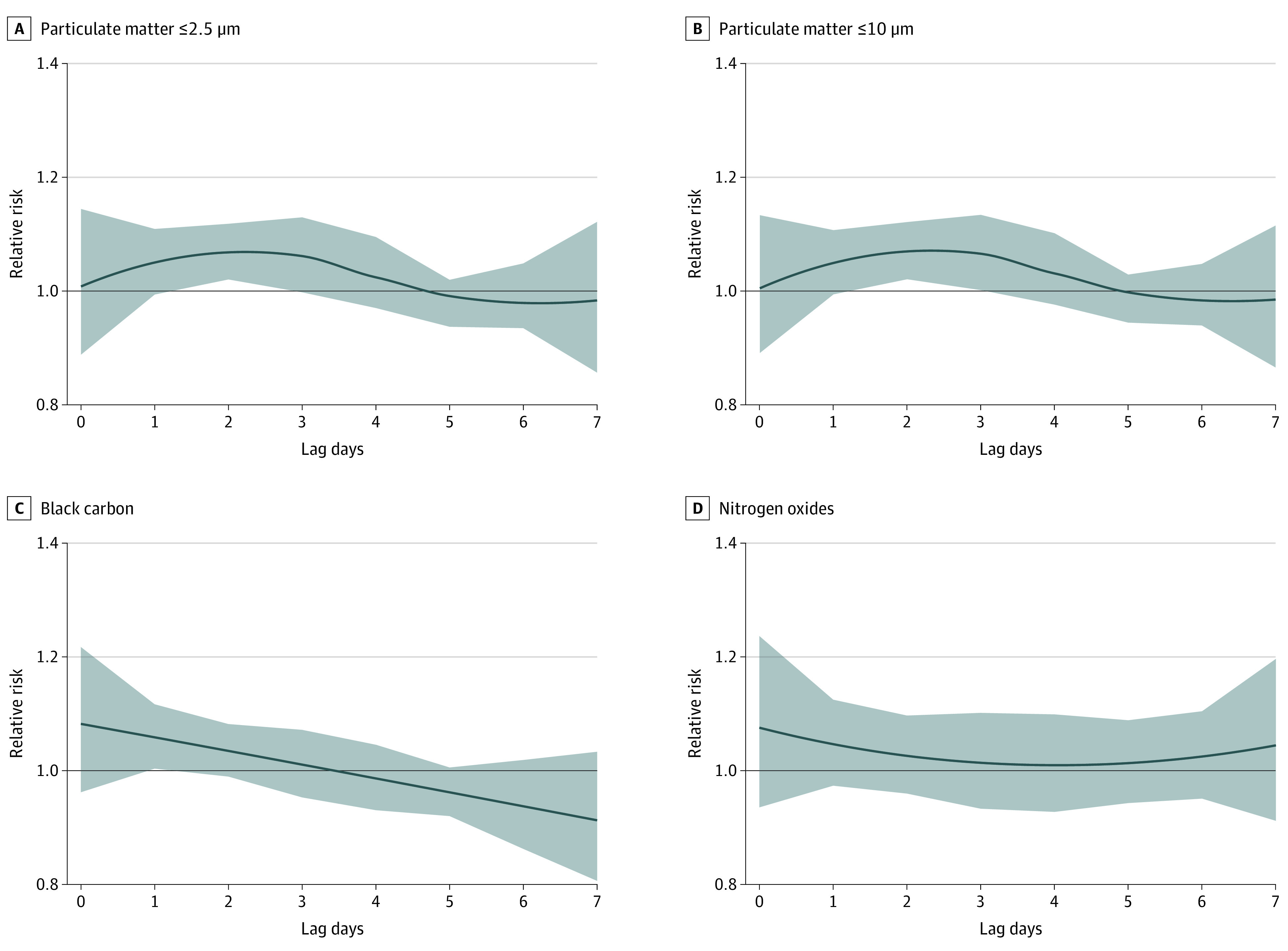
Lag-Specific Relative Risks (RRs) for SARS-CoV-2 Infection Associated With per-IQR Increase in Air Pollution Exposure A lag of 0 is the day of sampling for polymerase chain reaction testing (case days) and the days 7, 14, 21, and 28 days apart (control days), whereas a lag of 1 is the previous day and so forth. Curved line indicates RR; shaded areas, 95% CIs.

**Figure 2.**
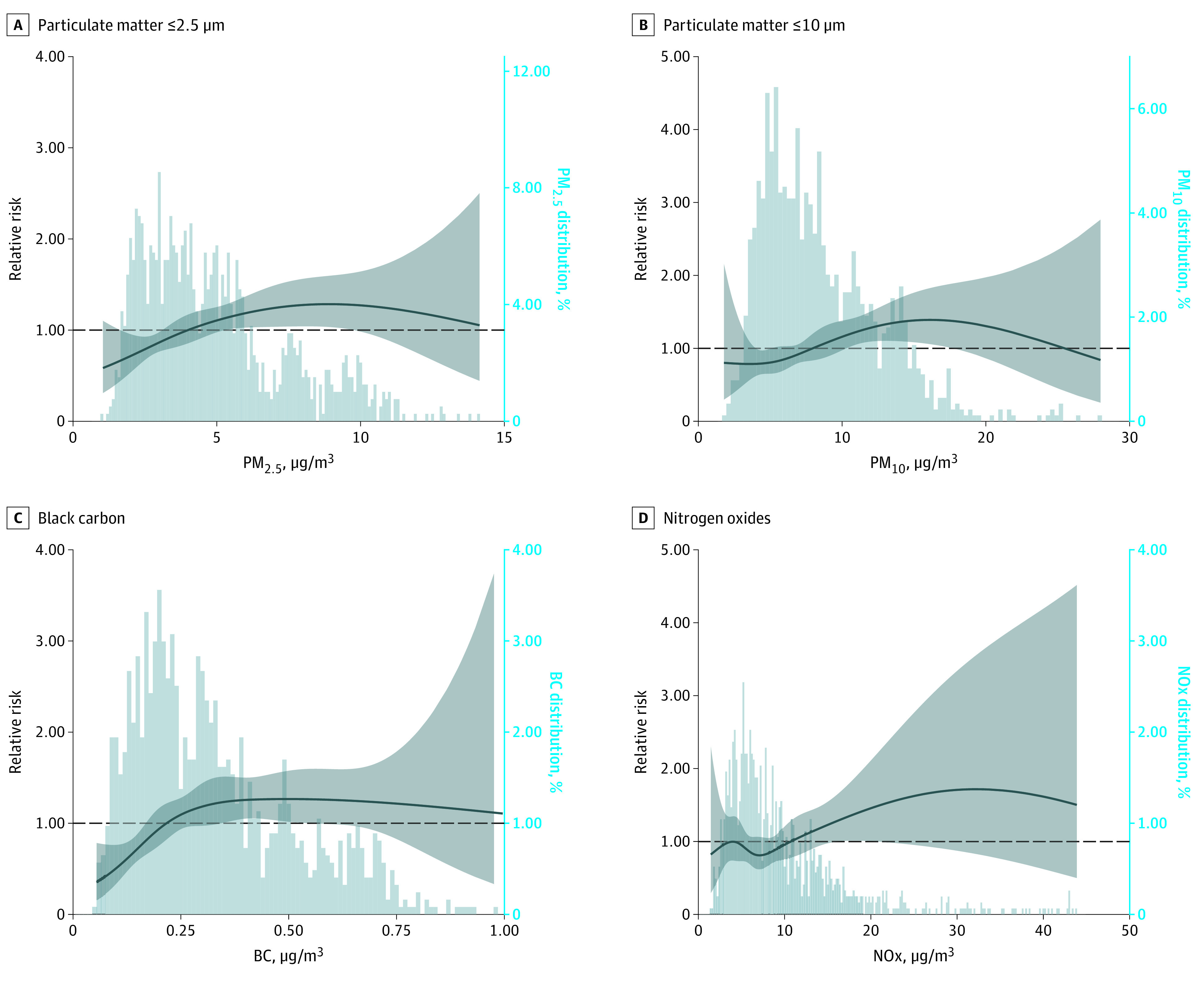
Exposure Response Curves Using Natural Splines With 3 Degrees of Freedom for the Association of Short-term Air Pollution Exposure and SARS-CoV-2 Infection A lag of 0 is the day of sampling for polymerase chain reaction testing (case days) and the days 7, 14, 21, and 28 days apart (control days), whereas a lag of 1 is the previous day and so forth. The exposure time window for air pollutants was lag 3 for particulate matter with diameter of 2.5 μm or less (PM_2.5_) and particulate matter with diameter of 10 μm or less (PM_10_), lag 1 for black carbon (BC), and lag 0 for nitrogen oxides (NOx). Curved line indicates RR; shaded areas, 95% CIs.

Figure 1 shows the lag-specific associations between SARS-CoV-2 infection and short-term exposure to air pollution. We observed associations at lag 2 for PM_10_ and PM_2.5_ (RR, 1.07 [95% CI, 1.02-1.12] for both) and at lag 1 for BC (RR, 1.06 [95% CI, 1.00-1.12]). The risk of having an infection increased by 6.9% (95% CI, 2.0%-12.1%) per IQR increase in PM_10_ exposure, by 6.8% (95% CI, 2.1%-11.8%) per IQR increase in PM_2.5_ exposure, and by 5.8% (95% CI, 0.3%-11.6%) per IQR increase in BC exposure (eTable 2 in Supplement 1 gives numeric results). We did not observe associations for NOx (RR, 1.05 [95% CI, 0.97-1.12] per IQR increase on lag 1). Using cumulative exposure generated a similar lag response but stronger effect size and wider 95% CIs (eTable 2 in Supplement 1). Extending the maximum lag to 14 days did not alter the results (eTable 3 in Supplement 1). No significant effect size modification was found in subgroup analyses (eFigures 3-14 in Supplement 1). Spline regression shows that increasing air pollution exposure was associated with increased risk of SARS-CoV-2 infection, with a steeper curve at lower levels of PM_2.5_, PM_10_, and BC. For NOx, the increasing trend was less informative owing to a small number of observations at high NOx exposure levels (Figure 2).

## Discussion

To our knowledge, this is the first report of individual-level, short-term exposure to air pollution associated with SARS-CoV-2 infection among young adults. Our case-crossover analysis based on data from a population-based cohort found an association between daily PM_2.5_, PM_10_, and BC exposure and a positive PCR test result (ie, having an infection). For each IQR increase in PM_2.5_, PM_10_, and BC daily concentrations, the risk increased significantly by approximately 6% to 7%. No significant interaction was found, suggesting a general association of acute air pollution exposure with SARS-CoV-2 infection.

Our results are consistent with those of previous ecological studies in several countries and regions indicating that areas with poorer air quality are more likely to have more infections,^33,34,35,36,37^ although the effect sizes and lag-time window of air pollution are heterogeneous between studies. A recent meta-analysis^12^ including 35 observational studies found that the COVID-19 incidence was associated with short-term exposure to PM_2.5_ (RR, 1.003 [95% CI, 1.002-1.004] per 1-μg/m^3^ increase) and PM_10_ (RR, 1.005 [95% CI, 1.003-1.008] per 1-μg/m^3^ increase), which is somewhat smaller than the effect size observed in the present study, although the differences in study design, target population, exposure distribution, and statistical methods preclude a direct comparison with our findings. Typically, previous ecological studies used a time-series study design and generalized additive model to quantify the association,^33,34,35,36,38,39,40^ which may be limited by the autocorrelation of air pollution concentrations over time and group- and population-level exposure. By using an individual-level case-crossover design and distributed-lag model, our study overcomes these limitations and shows an acute association of air pollution with SARS-CoV-2 infection. Moreover, our data add to the body of evidence that the association between air pollution and SARS-CoV-2 infection also exists in young adults because no previous short-term studies focused on this subpopulation or reported interaction by age.

In this study, we observed a shorter lag-response association between air pollution and SARS-CoV-2 infection (the association of PM_2.5_ and PM_10_ exposure peaks on lag day 2, and BC exposure peaks on lag day 1) because the median incubation period was approximately 5 days.^41^ In addition, recent literature suggests that transmission of SARS-CoV-2 is more likely to be related to indoor settings rather than the outdoor environment.^16^ In Wuhan, China, a study found that only 2 of 7324 COVID-19 cases could be linked to transmission in an outdoor environment.^42^ In Ireland, only 262 of 232 164 cases were linked to transmission in outdoor environment reported by the Health Protection Surveillance Centre.^43^ In Italy, a study found that the viral particle concentrations were very low in ambient air samples.^44^ Taken together, we speculate that increased levels of short-term air pollution play a role in manifesting the disease (symptoms) for those who have been infected with the virus rather than contributing to the transmission of the virus. Short-term exposure to air pollution can affect airway inflammation and oxidative stress,^8,45^ whereas absorbed air pollutants may cause deep lung irritation and immunomodulation of the host response to infection, possibly worsening the severity of existing infection.^46^ Previously, Kogevinas et al^47^ also found that long-term exposure to air pollution was not associated with SARS-CoV-2 infection (measured by antibody levels in blood samples) per se but was associated with severity after infection (based on hospitalizations and self-reported symptoms) in a Spanish cohort. More mechanistic studies are needed to examine this hypothesis.^48^

We did not observe any interaction of the observed association between air pollution and SARS-CoV-2 infection by sex, smoking, or having overweight, asthma, or COVID-19–related respiratory symptoms. This outcome could be explained in part by the general effects of ambient air pollution. However, the present outcome based on registry data was influenced by test-seeking behaviors, whereas a large proportion of young adults were asymptomatic or with mild symptoms after infection, and therefore the included participants with infection were not representative of the general young adult population. Outcome assessment that can capture all individuals with current or past infection (such as measurement of antibody levels) is needed to investigate the potential effect size modifier in the future study.

### Strengths and Limitations

The main strengths of our study include the time-stratified case-crossover design that controlled for time-invariant confounding factors (eg, population density, lifestyle factors), ascertainment of cases based on PCR testing results from the national register of infectious diseases, application of a distributed-lag model that assesses the acute and delayed effect size of exposure, use of high-resolution spatiotemporal air pollution modeling to estimate exposure on an individual level, and ability to perform subgroup analysis based on selected characteristics. Potential limitations include exposure misclassification given that we estimated exposure to outdoor air pollution, whereas information on microclimate differences in exposure or time-activity patterns (eg, time spent in traffic and indoors) was not available. In addition, we investigated predisposition factors in a relatively small group of participants with only mild to moderate disease,^20^ which limited the statistical power. Owing to the high correlations between air pollutants, we did not test the 2-pollutant model to assess the independence of each pollutant. We were also unable to exclude the existence of residual time-varying confounding factors. Further individual-level studies with a larger sample size, preferably from different geographical regions, are needed to verify the association between short-term air pollution and SARS-CoV-2 infection.

## Conclusions

The findings of this case-crossover study of Swedish young adults with air pollution exposure and SARS-CoV-2 infection suggest that residential short-term exposure to air pollution was associated with increased risk of having positive PCR test results for SARS-CoV-2 infection. These findings support the broad public health benefits of reducing ambient air pollution levels.

## Notes

Supplement 1. **eTable 1.** Characteristics of BAMSE Subjects Identified as SARS-CoV-2 Infection Cases From SmiNet, Participants of COVID-19 Follow-up, and 24-Year Follow-up **eTable 2.** Lag-Specific Relative Risks for SARS-CoV-2 Infection Associated With per-IQR Increase in Short-term Air Pollution Exposure Using Single-Day Lag and Cumulative Lag **eTable 3.** Lag-Specific Relative Risks for SARS-CoV-2 Infection Associated With per-IQR Increase in Short-term Air Pollution Exposure From Lag 0 to Lag 14 **eFigure 1.** Daily Variation of Modelled Air Pollutants and Observed Temperature at the SITE of the Urban Background Station in Central Stockholm Torkel Knutssonsgatan During the Study Period **eFigure 2.** Correlation (Spearman) Matrix Between Air Pollutants and Temperature in Different Time Windows **eFigure 3.** Lag-Specific Relative Risks for SARS-CoV-2 Infection Associated With per-IQR Increase in Short-term Air Pollution Exposure Stratified by Sex **eFigure 4.** Lag-Specific Relative Risks for SARS-CoV-2 Infection Associated With per-IQR Increase in Short-term Air Pollution Exposure Stratified by Asthma **eFigure 5.** Lag-Specific Relative Risks for SARS-CoV-2 Infection Associated With per-IQR Increase in Short-term Air Pollution Exposure Stratified by Smoking Status **eFigure 6.** Lag-Specific Relative Risks for SARS-CoV-2 Infection Associated With per-IQR Increase in Short-term Air Pollution Exposure Stratified by Having Overweight **eFigure 7.** Lag-Specific Relative Risks for SARS-CoV-2 Infection Associated With per-IQR Increase in Short-term Air Pollution Exposure Stratified by Season **eFigure 8.** Lag-Specific Relative Risks for SARS-CoV-2 Infection Associated With per-IQR Increase in Short-term Air Pollution Exposure Stratified by Self-reported Fever **eFigure 9.** Lag-Specific Relative Risks for SARS-CoV-2 Infection Associated With per-IQR Increase in Short-term Air Pollution Exposure Stratified by Self-reported Cough **eFigure 10.** Lag-Specific Relative Risks for SARS-CoV-2 Infection Associated With per-IQR Increase in Short-term Air Pollution Exposure Stratified by Self-reported Sore Throat **eFigure 11.** Lag-Specific Relative Risks for SARS-CoV-2 Infection Associated With per-IQR Increase in Short-term Air Pollution Exposure Stratified by Self-reported Sense of Taste and Smell **eFigure 12.** Lag-Specific Relative Risks for SARS-CoV-2 Infection Associated With per-IQR Increase in Short-term Air Pollution Exposure Stratified by Self-reported Nasal Congestion **eFigure 13.** Lag-Specific Relative Risks for SARS-CoV-2 Infection Associated With per-IQR Increase in Short-term Air Pollution Exposure Stratified by Self-reported Sniffle **eFigure 14.** Lag-Specific Relative Risks for SARS-CoV-2 Infection Associated With per-IQR Increase in Short-term Air Pollution Exposure Stratified by Self-reported Breathing Difficulty

Supplement 2.
Nonauthor Collaborators
