# Supplementary material for: Association of Short-term Air Pollution Exposure With SARS-CoV-2 Infection Among Young Adults in Sweden
Source: JAMA Netw Open. 2022 Apr 20;5(4):e228109. doi: 10.1001/jamanetworkopen.2022.8109 (PMC9021914; doi:10.1001/jamanetworkopen.2022.8109)
Supplement: Supplement 1. — eTable 1. Characteristics of BAMSE Subjects Identified as SARS-CoV-2 Infection Cases From SmiNet, Participants of COVID-19 Follow-up, and 24-Year Follow-up eTable 2. Lag-Specific Relative Risks for SARS-CoV-2 Infection Associated With per-IQR Increase in Short-term Air Pollution Exposure Using Single-Day Lag and Cumulative Lag eTable 3. Lag-Specific Relative Risks for SARS-CoV-2 Infection Associated With per-IQR Increase in Short-term Air Pollution Exposure From Lag 0 to Lag 14 eFigure 1. Daily Variation of Modelled Air Pollutants and Observed Temperature at the SITE of the Urban Background Station in Central Stockholm Torkel Knutssonsgatan During the Study Period eFigure 2. Correlation (Spearman) Matrix Between Air Pollutants and Temperature in Different Time Windows eFigure 3. Lag-Specific Relative Risks for SARS-CoV-2 Infection Associated With per-IQR Increase in Short-term Air Pollution Exposure Stratified by Sex eFigure 4. Lag-Specific Relative Risks for SARS-CoV-2 Infection Associated With per-IQR Increase in Short-term Air Pollution Exposure Stratified by Asthma eFigure 5. Lag-Specific Relative Risks for SARS-CoV-2 Infection Associated With per-IQR Increase in Short-term Air Pollution Exposure Stratified by Smoking Status eFigure 6. Lag-Specific Relative Risks for SARS-CoV-2 Infection Associated With per-IQR Increase in Short-term Air Pollution Exposure Stratified by Having Overweight eFigure 7. Lag-Specific Relative Risks for SARS-CoV-2 Infection Associated With per-IQR Increase in Short-term Air Pollution Exposure Stratified by Season eFigure 8. Lag-Specific Relative Risks for SARS-CoV-2 Infection Associated With per-IQR Increase in Short-term Air Pollution Exposure Stratified by Self-reported Fever eFigure 9. Lag-Specific Relative Risks for SARS-CoV-2 Infection Associated With per-IQR Increase in Short-term Air Pollution Exposure Stratified by Self-reported Cough eFigure 10. Lag-Specific Relative Risks for SARS-CoV-2 Infection Associated With per-IQR Incre [file jamanetwopen-e228109-s001.pdf]

Supplementary Online Content

Yu Z, Bellander T, Bergström A, et al; BAMSE COVID-19 Study Group. Association of short-term air pollution exposure with SARS-CoV-2 infection among young adults in Sweden. *JAMA Netw Open*. 2022;5(4):e228109. doi:10.1001/jamanetworkopen.2022.8109

eTable 1. Characteristics of BAMSE Subjects Identified as SARS-CoV-2 Infection Cases From SmiNet, Participants of COVID-19 Follow-up, and 24-Year Follow-up
eTable 2. Lag-Specific Relative Risks for SARS-CoV-2 Infection Associated With per-IQR Increase in Short-term Air Pollution Exposure Using Single-Day Lag and Cumulative Lag
eTable 3. Lag-Specific Relative Risks for SARS-CoV-2 Infection Associated With per-IQR Increase in Short-term Air Pollution Exposure From Lag 0 to Lag 14
eFigure 1. Daily Variation of Modelled Air Pollutants and Observed Temperature at the SITE of the Urban Background Station in Central Stockholm Torkel Knutssonsgatan During the Study Period
eFigure 2. Correlation (Spearman) Matrix Between Air Pollutants and Temperature in Different Time Windows
eFigure 3. Lag-Specific Relative Risks for SARS-CoV-2 Infection Associated With per-IQR Increase in Short-term Air Pollution Exposure Stratified by Sex
eFigure 4. Lag-Specific Relative Risks for SARS-CoV-2 Infection Associated With per-IQR Increase in Short-term Air Pollution Exposure Stratified by Asthma
eFigure 5. Lag-Specific Relative Risks for SARS-CoV-2 Infection Associated With per-IQR Increase in Short-term Air Pollution Exposure Stratified by Smoking Status
eFigure 6. Lag-Specific Relative Risks for SARS-CoV-2 Infection Associated With per-IQR Increase in Short-term Air Pollution Exposure Stratified by Having Overweight
eFigure 7. Lag-Specific Relative Risks for SARS-CoV-2 Infection Associated With per-IQR Increase in Short-term Air Pollution Exposure Stratified by Season
eFigure 8. Lag-Specific Relative Risks for SARS-CoV-2 Infection Associated With per-IQR Increase in Short-term Air Pollution Exposure Stratified by Self-reported Fever
eFigure 9. Lag-Specific Relative Risks for SARS-CoV-2 Infection Associated With per-IQR Increase in Short-term Air Pollution Exposure Stratified by Self-reported Cough
eFigure 10. Lag-Specific Relative Risks for SARS-CoV-2 Infection Associated With per-IQR Increase in Short-term Air Pollution Exposure Stratified by Self-reported Sore Throat
eFigure 11. Lag-Specific Relative Risks for SARS-CoV-2 Infection Associated With per-IQR Increase in Short-term Air Pollution Exposure Stratified by Self-reported Sense of Taste and Smell
eFigure 12. Lag-Specific Relative Risks for SARS-CoV-2 Infection Associated With per-IQR Increase in Short-term Air Pollution Exposure Stratified by Self-reported Nasal Congestion
eFigure 13. Lag-Specific Relative Risks for SARS-CoV-2 Infection Associated With per-IQR Increase in Short-term Air Pollution Exposure Stratified by Self-reported Sniffle

© 2022 Yu Z et al. *JAMA Network Open*.

eFigure 14. Lag-Specific Relative Risks for SARS-CoV-2 Infection Associated With per-IQR Increase in Short-term Air Pollution Exposure Stratified by Self-reported Breathing Difficulty

This supplementary material has been provided by the authors to give readers additional information about their work.

© 2022 Yu Z et al. *JAMA Network Open*.

eTable 1. Characteristics of BAMSE Subjects Identified as SARS-CoV-2 Infection Cases From SmiNet, Participants of COVID-19 Follow-up, and 24-Year Follow-up

	Participants identified from SmiNet (N=425)	BAMSE COVID-19 follow-up phase 1 (N=1645)	BAMSE 24-year follow-up (N=2270)
**Basic characteristics**			
**Female sex**	229/425 (53.9)	996/1645 (60.6)	1266/2270 (55.8)
**Education**			
University	115/345 (33.3)	641/1638 (39.1)	859/2261 (38.0)
Elementary or High school	230/345 (66.7)	997/1638 (60.9)	1402/2261 (62.0)
**Occupation**			
Study	164/345 (47.6)	616/1638 (37.6)	1201/2267 (52.9)
Employed	154/345 (44.6)	844/1638 (51.5)	890/2267 (39.3)
Other	27/345 (7.8)	178 /1638 (10.9)	176/2267 (7.8)
**Overweight**	61/278 (21.9)	349/1638 (21.3)	517/2270 (22.8)
**Current smoking**	79/345 (22.9)	242/1641 (14.7)	457/2266 (20.2)
**Asthma**	126/408 (30.9)	511/1314 (38.9)	946/2270 (41.7)

© 2022 Yu Z et al. *JAMA Network Open*.

**COVID related characteristics**			
**Any symptoms (any of the below)**	107/200 (53.5)	742/1640 (45.2)	NA
**Type of respiratory symptoms**			
Fever	76/144 (52.8)	462/712 (64.9)	NA
Cough	81/145 (55.9)	488/737 (66.2)	NA
Sore throat	78/144 (54.2)	523/734 (71.2)	NA
Loss of taste or smell	70/146 (48.0)	271/732 (38.6)	NA
Sniffles	115/146 (78.8)	532/731 (72.8)	NA
Nasal congestions	96/146 (65.8)	457/726 (63.0)	NA
Breathing difficulties	37/145 (25.5)	255/732 (34.8)	NA
**COVID cases in household**	81/142 (57.0)	484/1643 (29.5)	NA
**Regular meeting people during pandemic**	147/200 (73.5)	641/1638 (39.1)	NA
**Use of public transportation during pandemic**	62/200 (31.0)	435/1638 (26.6)	NA

Data are presented as n/N (%). NA: not applicable.

© 2022 Yu Z et al. *JAMA Network Open*.

eTable 2. Lag-Specific Relative Risks for SARS-CoV-2 Infection Associated With per-IQR Increase in Short-term Air Pollution Exposure Using Single-Day Lag and Cumulative Lag

Exposure window	RR (95%CI)			
	$PM_{2.5}$	PM_{10}	BC	NO_x
**Single day lag**				
Lag 0	1.01 (0.89, 1.14)	1.00 (0.89, 1.13)	1.08 (0.96, 1.22)	1.08 (0.94, 1.24)
Lag 1	1.05 (0.99, 1.11)	1.05 (0.99, 1.11)	1.06 (1.00, 1.12)	1.05 (0.97, 1.12)
Lag 2	1.07 (1.02, 1.12)	1.07 (1.02, 1.12)	1.03 (0.99, 1.08)	1.03 (0.96, 1.10)
Lag 3	1.06 (1.00, 1.13)	1.06 (1.00, 1.13)	1.01 (0.95, 1.07)	1.01 (0.93, 1.10)
Lag 4	1.03 (0.97, 1.10)	1.04 (0.98, 1.10)	0.99 (0.93, 1.05)	1.01 (0.93, 1.10)
Lag 5	0.98 (0.94, 1.02)	0.98 (0.94, 1.03)	0.96 (0.92, 1.01)	1.01 (0.94, 1.09)
Lag 6	0.99 (0.93, 1.05)	0.99 (0.94, 1.05)	0.94 (0.86, 1.02)	1.02 (0.95, 1.10)
Lag 7	0.98 (0.86, 1.12)	0.98 (0.86, 1.11)	0.91 (0.81, 1.03)	1.04 (0.91, 1.20)
**Cumulative lag**				
Lag 01	1.08 (0.92, 1.29)	1.09 (0.92, 1.29)	1.13 (0.96, 1.34)	1.04 (0.84, 1.28)
Lag 02	1.15 (0.97, 1.38)	1.17 (0.98, 1.40)	1.08 (0.93, 1.24)	1.11 (0.88, 1.39)
Lag 03	1.16 (0.97, 1.38)	1.20 (0.99, 1.44)	1.04 (0.91, 1.19)	1.08 (0.84, 1.38)
Lag 04	1.06 (0.86, 1.30)	1.07 (0.88, 1.31)	1.06 (0.94, 1.20)	1.07 (0.81, 1.40)
Lag 05	0.97 (0.89, 1.19)	1.08 (0.90, 1.31)	1.04 (0.90, 1.22)	1.09 (0.82, 1.44)
Lag 06	0.99 (0.84, 1.19)	1.01 (0.83, 1.22)	0.96 (0.82, 1.12)	1.13 (0.84, 1.53)
Lag 07	0.93 (0.78, 1.10)	0.92 (0.76, 1.12)	0.96 (0.82, 1.12)	1.13 (0.82, 1.55)

PM10: particulate matter with diameter less than 10 µm; PM2.5, particulate matter with diameter less than 2.5 µm; BC, black carbon; NOx, nitrogen oxides. A lag of zero is the day of PCR-test sampling, while a lag of one is the day before the sampling**.**

© 2022 Yu Z et al. *JAMA Network Open*.

eTable 3. Lag-Specific Relative Risks for SARS-CoV-2 Infection Associated With per-IQR Increase in Short-term Air Pollution Exposure From Lag 0 to Lag 14

Lag days	RR (95%CIs)			
	$PM_{2.5}$	PM_{10}	BC	NOx
Lag 0	1.01 (0.89, 1.14)	1.00 (0.89, 1.13)	1.09 (0.78, 1.52)	1.03 (0.94,1.13)
Lag 1	1.05 (0.99, 1.11)	1.05 (0.99, 1.11)	1.07 (0.79, 1.45)	1.03 (0.95,1.12)
Lag 2	1.07 (1.02, 1.12)	1.07 (1.02, 1.12)	1.05 (0.79, 1.39)	1.04 (0.96,1.12)
Lag 3	1.06 (1.00, 1.13)	1.06 (1.00, 1.13)	1.03 (0.80, 1.33)	1.04 (0.97,1.12)
Lag 4	1.03 (0.97, 1.10)	1.04 (0.98, 1.10)	1.01 (0.80, 1.28)	1.05 (0.98,1.12)
Lag 5	0.98 (0.94, 1.02)	0.98 (0.94, 1.03)	0.99 (0.79, 1.23)	1.05 (0.99,1.12)
Lag 6	0.99 (0.93, 1.05)	0.99 (0.94, 1.05)	0.97 (0.78, 1.20)	1.05 (0.98,1.14)
Lag 7	0.98 (0.86, 1.12)	0.98 (0.86, 1.11)	0.95 (0.77, 1.17)	1.06 (0.97,1.15)
Lag 8	0.97 (0.89, 1.15)	1.00 (0.96, 1.04)	0.93 (0.76, 1.14)	1.06 (0.98,1.14)
Lag 9	0.98 (0.93, 1.03)	1.00 (0.96, 1.05)	0.91 (0.74, 1.13)	1.07 (0.99,1.14)
Lag 10	1.00 (0.96, 1.04)	1.00 (0.95, 1.05)	0.89 (0.72, 1.12)	1.07 (0.98,1.17)
Lag 11	1.00 (0.96, 1.04)	1.00 (0.95, 1.05)	0.88 (0.69, 1.11)	1.08 (0.98,1.17)
Lag 12	0.99 (0.96, 1.03)	0.99 (0.95, 1.04)	0.86 (0.66, 1.11)	1.08 (0.97,1.19)
Lag 13	0.99 (0.96, 1.03)	0.98 (0.94, 1.03)	0.84 (0.64, 1.11)	1.08 (0.99,1.18)
Lag 14	0.99 (0.97, 1.02)	0.97 (0.89, 1.06)	0.83 (0.61, 1.12)	1.09 (0.98,1.20)

PM10: particulate matter with diameter less than 10 µm; PM2.5, particulate matter with diameter less than 2.5 µm; BC, black carbon; NOx, nitrogen oxides. A lag of zero is the day of PCR-test sampling, while a lag of one is the day before the sampling.

© 2022 Yu Z et al. *JAMA Network Open*.

eFigure 1. Daily Variation of Modelled Air Pollutants and Observed Temperature at the SITE of the Urban Background Station in Central Stockholm Torkel Knutssonsgatan During the Study Period

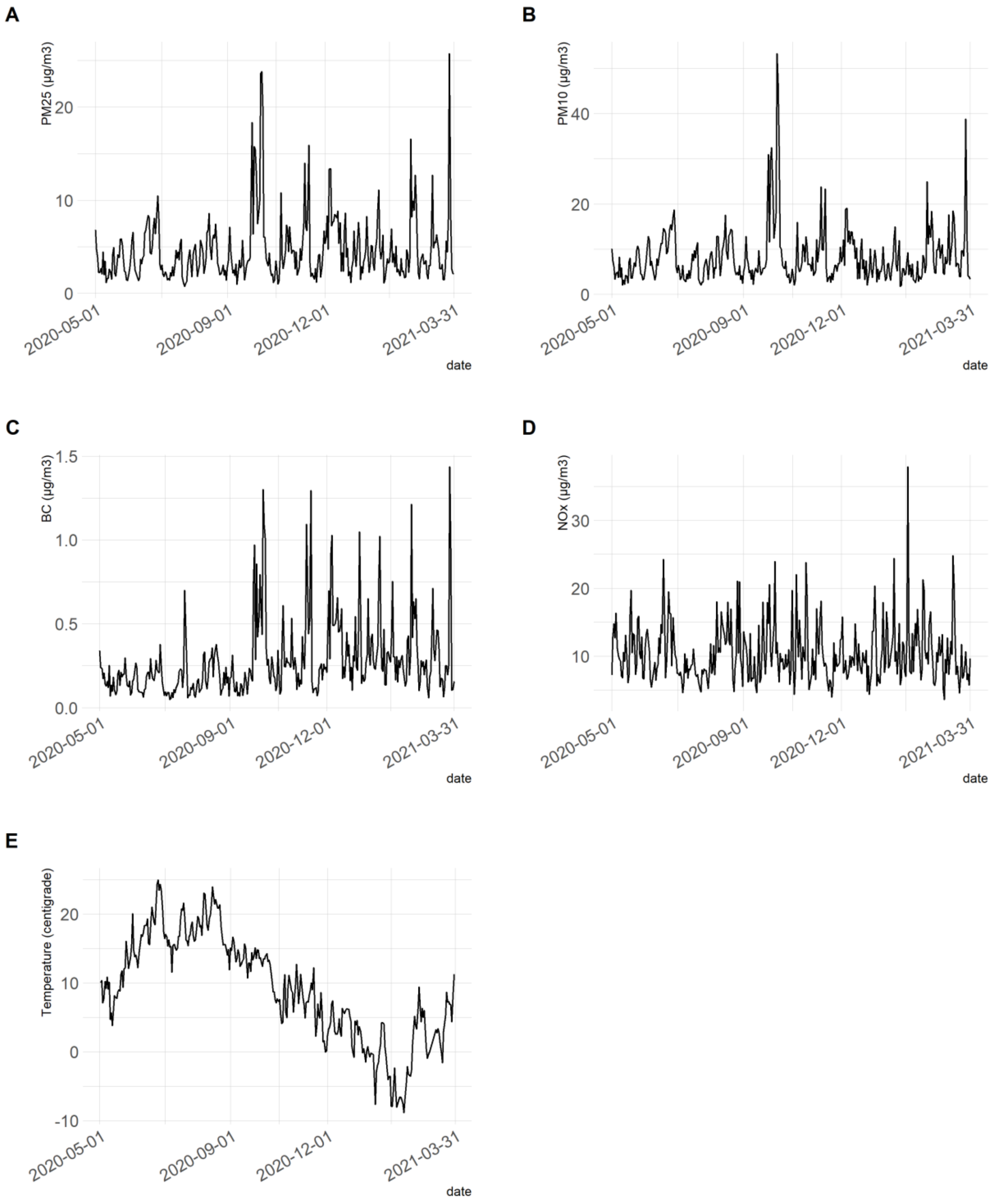

A. particular matter with diameter less than 2.5 µm; B. particular matter with diameter less than 10 µm; C. black carbon; D. nitrogen oxides. E. Temperature.

© 2022 Yu Z et al. *JAMA Network Open*.

eFigure 2. Correlation (Spearman) Matrix Between Air Pollutants and Temperature in Different Time Windows

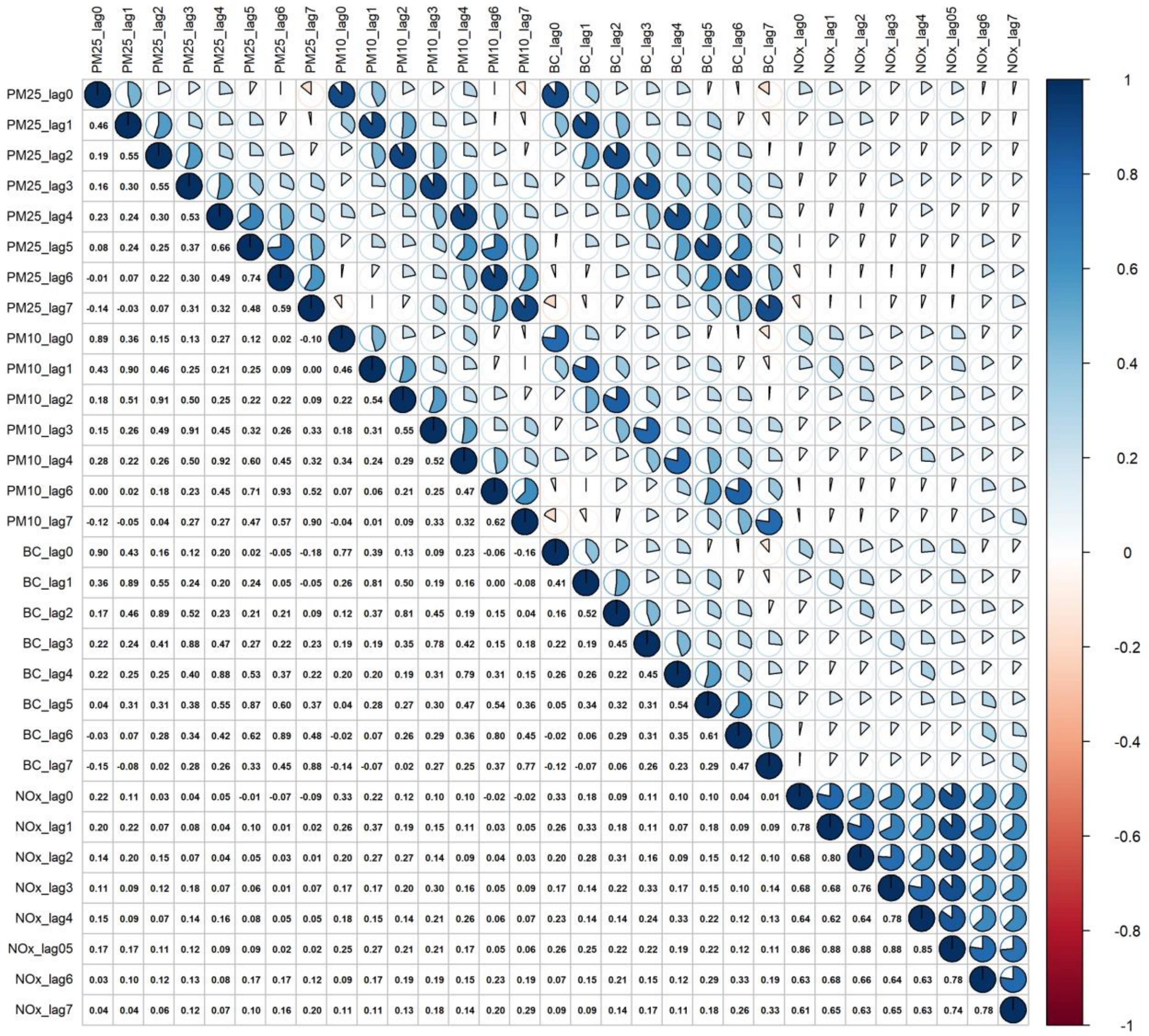

© 2022 Yu Z et al. *JAMA Network Open*.

eFigure 3. Lag-Specific Relative Risks for SARS-CoV-2 Infection Associated With per-IQR Increase in Short-term Air Pollution Exposure Stratified by Sex

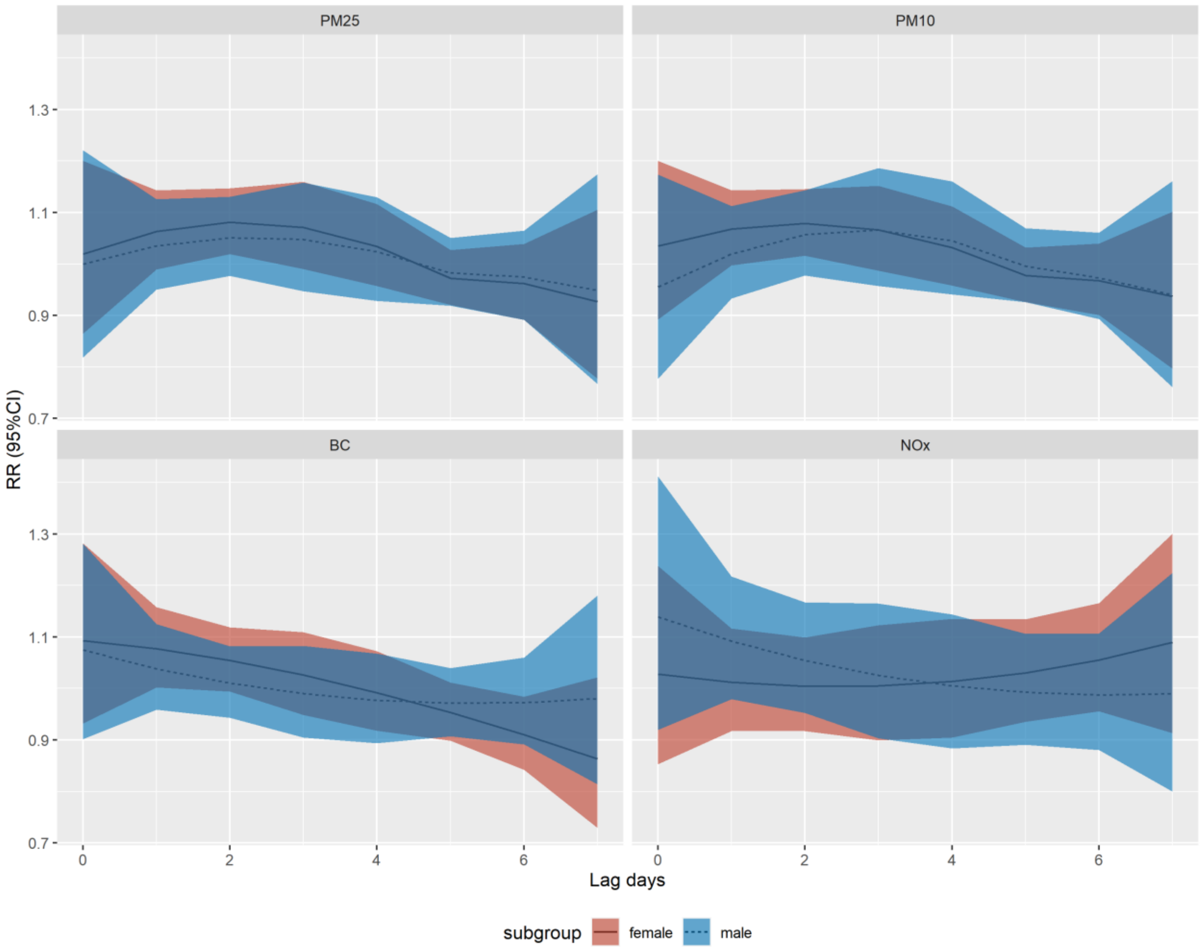

© 2022 Yu Z et al. *JAMA Network Open*.

eFigure 4. Lag-Specific Relative Risks for SARS-CoV-2 Infection Associated With per-IQR Increase in Short-term Air Pollution Exposure Stratified by Asthma

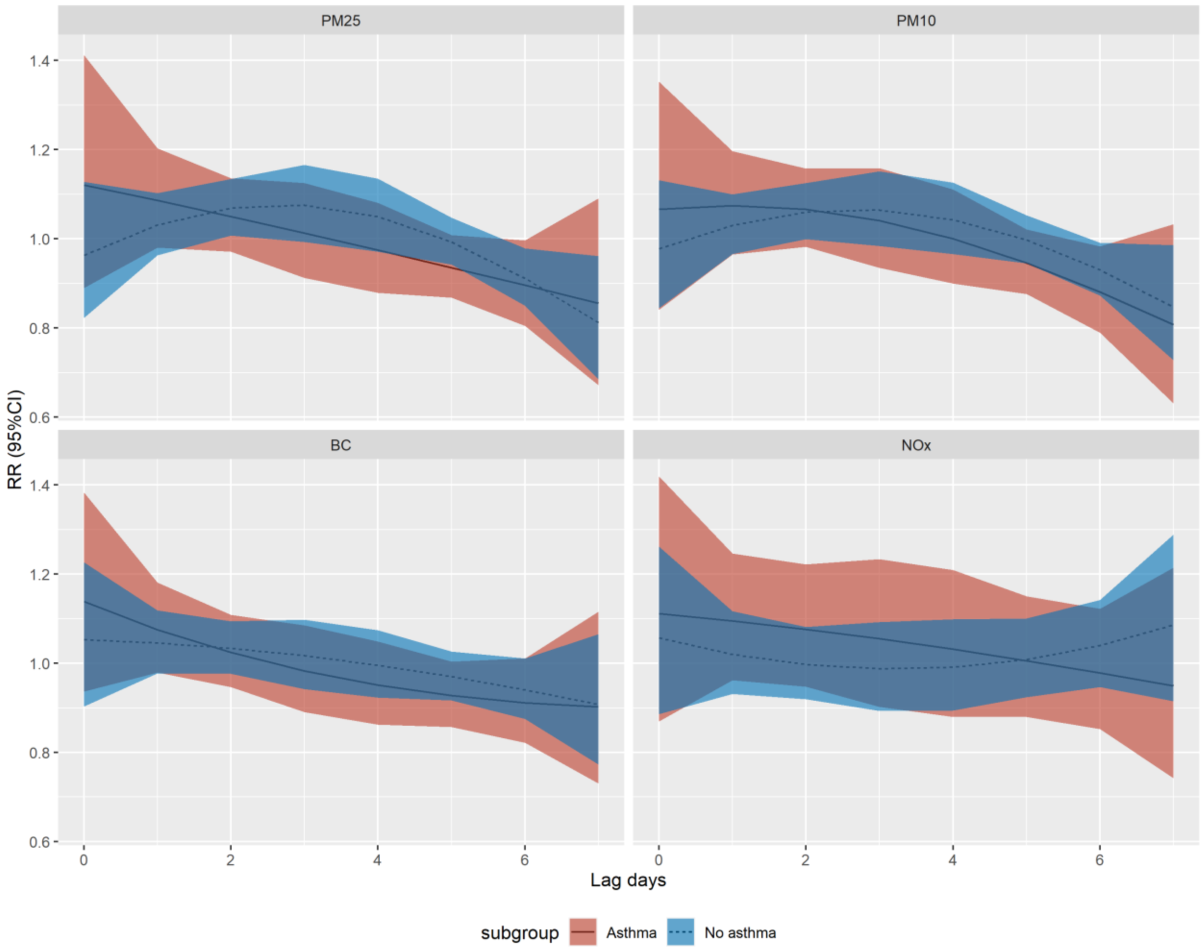

© 2022 Yu Z et al. *JAMA Network Open*.

eFigure 5. Lag-Specific Relative Risks for SARS-CoV-2 Infection Associated With per-IQR Increase in Short-term Air Pollution Exposure Stratified by Smoking Status

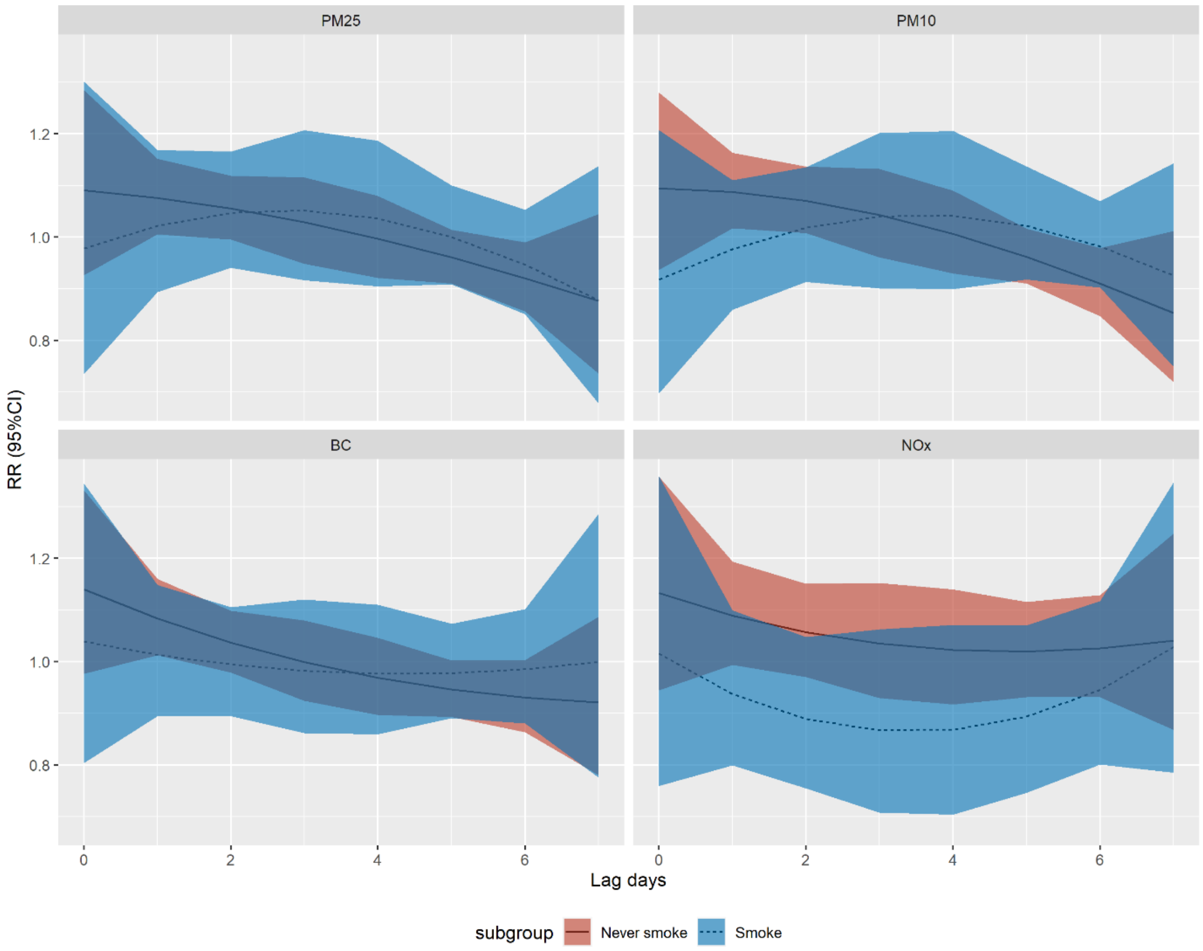

© 2022 Yu Z et al. *JAMA Network Open*.

eFigure 6. Lag-Specific Relative Risks for SARS-CoV-2 Infection Associated With per-IQR Increase in Short-term Air Pollution Exposure Stratified by Having Overweight

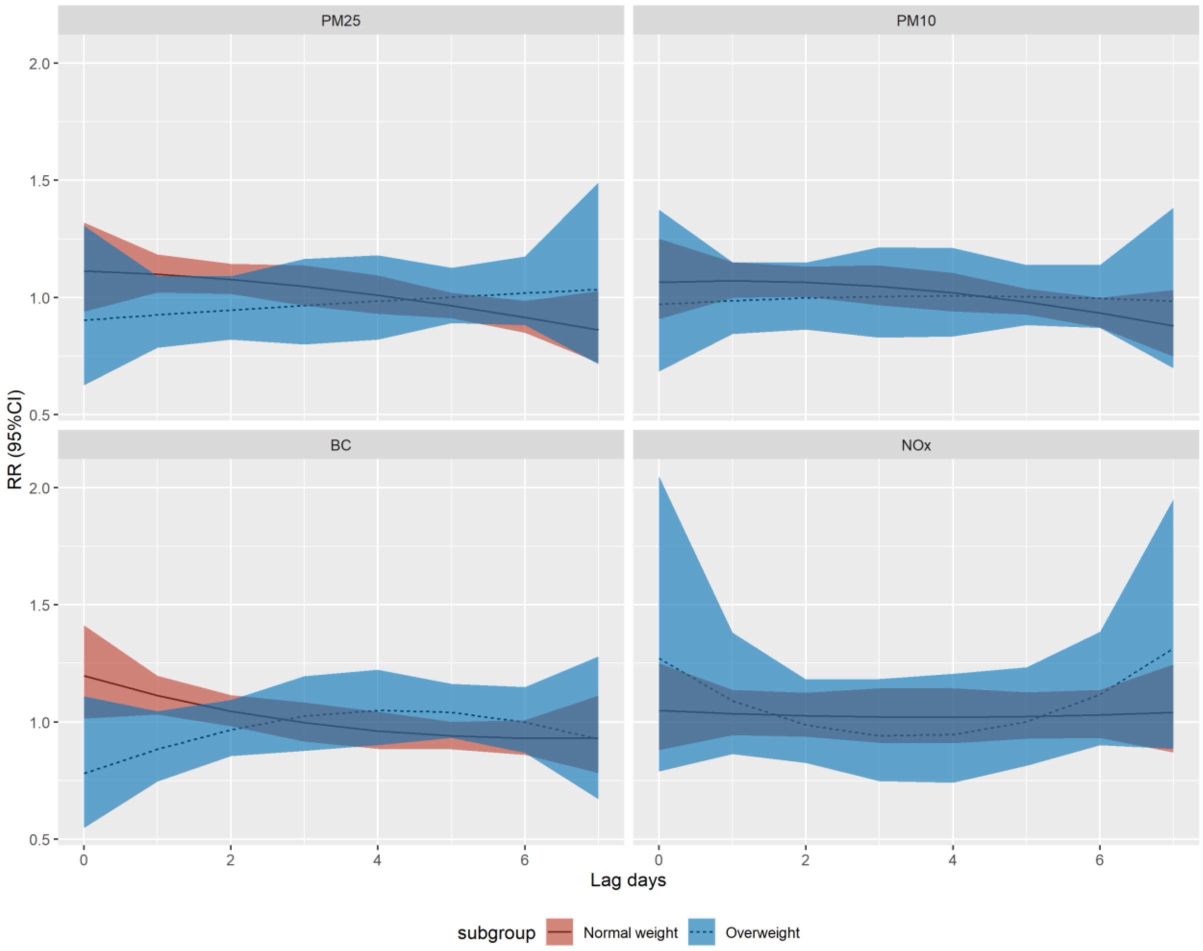

© 2022 Yu Z et al. *JAMA Network Open*.

eFigure 7. Lag-Specific Relative Risks for SARS-CoV-2 Infection Associated With per-IQR Increase in Short-term Air Pollution Exposure Stratified by Season

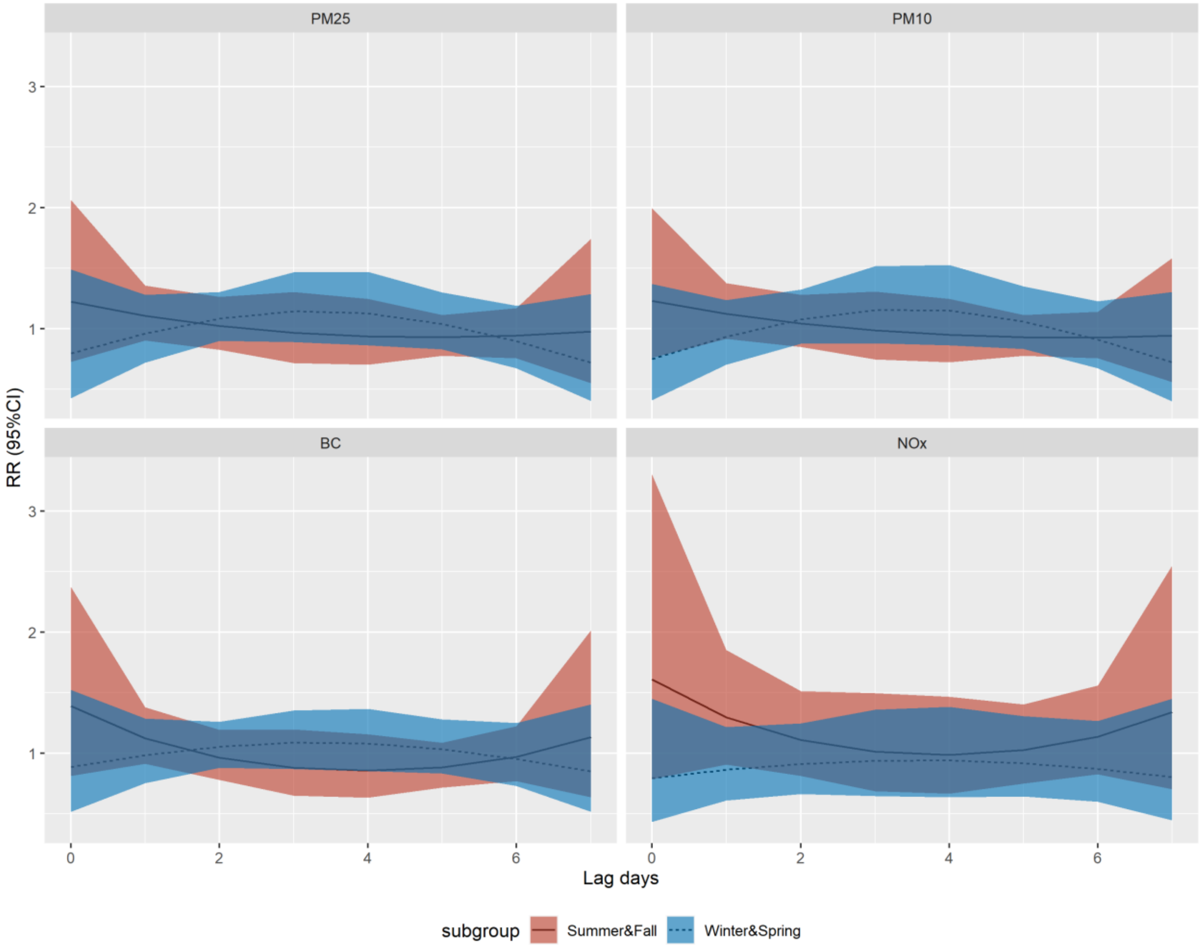

© 2022 Yu Z et al. *JAMA Network Open*.

eFigure 8. Lag-Specific Relative Risks for SARS-CoV-2 Infection Associated With per-IQR Increase in Short-term Air Pollution Exposure Stratified by Self-reported Fever

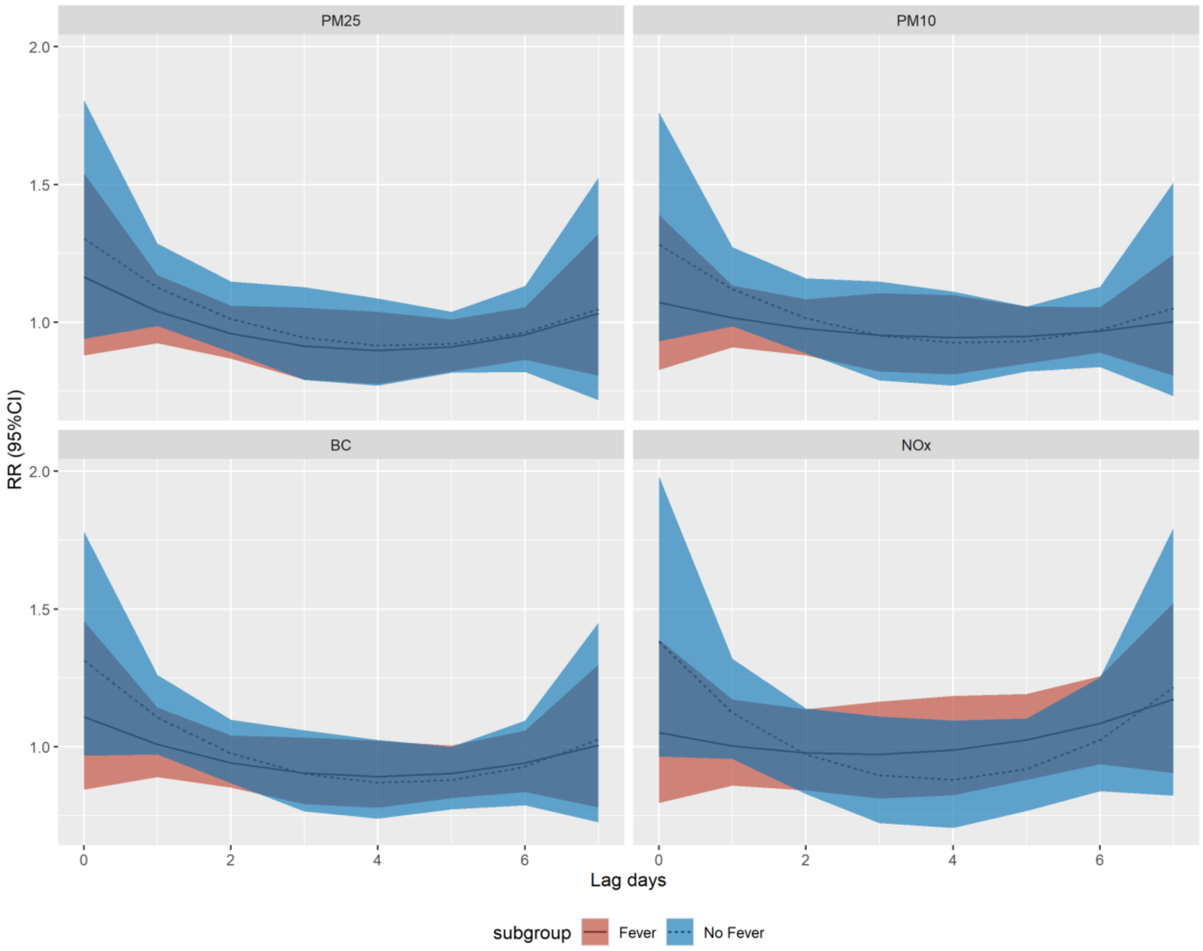

© 2022 Yu Z et al. *JAMA Network Open*.

eFigure 9. Lag-Specific Relative Risks for SARS-CoV-2 Infection Associated With per-IQR Increase in Short-term Air Pollution Exposure Stratified by Self-reported Cough

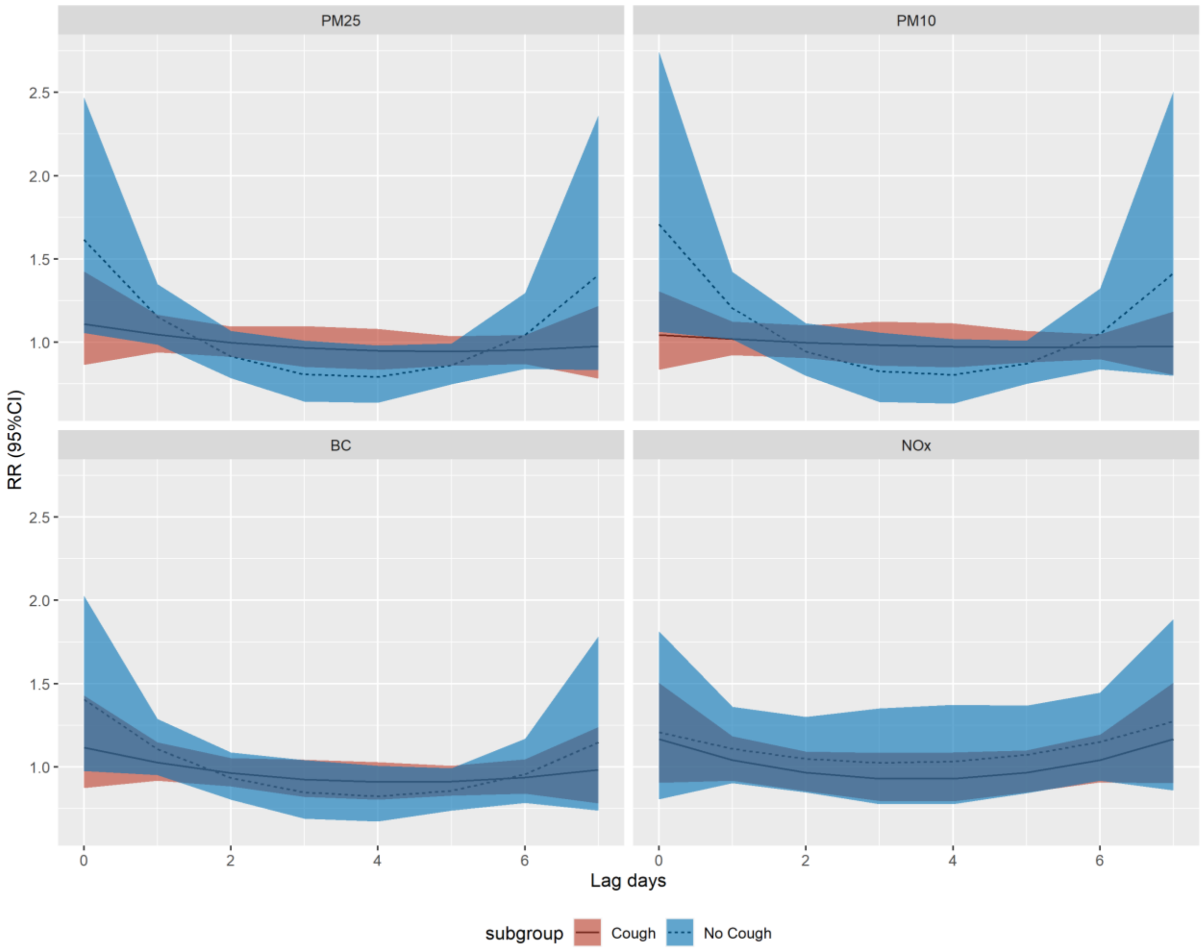

© 2022 Yu Z et al. *JAMA Network Open*.

eFigure 10. Lag-Specific Relative Risks for SARS-CoV-2 Infection Associated With per-IQR Increase in Short-term Air Pollution Exposure Stratified by Self-reported Sore Throat

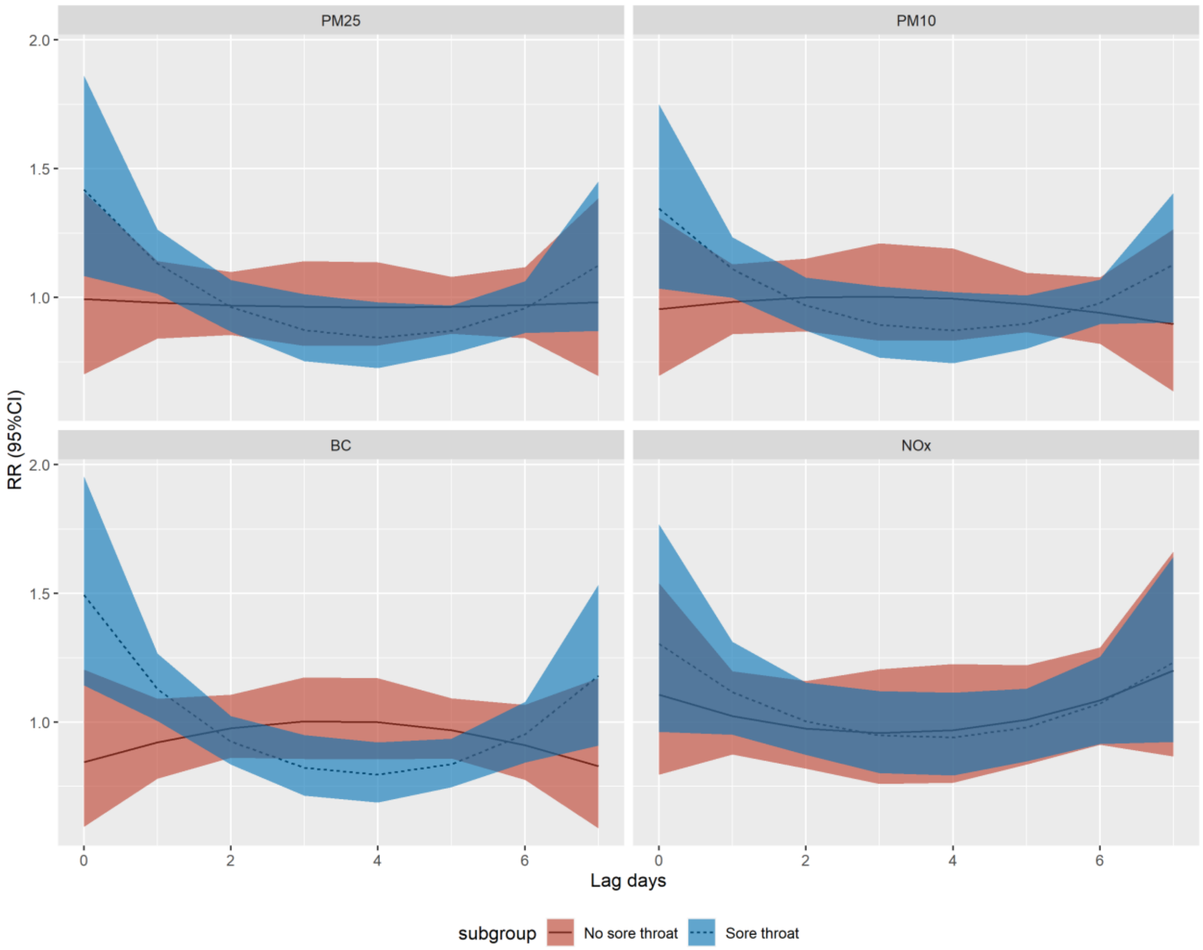

© 2022 Yu Z et al. *JAMA Network Open*.

eFigure 11. Lag-Specific Relative Risks for SARS-CoV-2 Infection Associated With per-IQR Increase in Short-term Air Pollution Exposure Stratified by Self-reported Sense of Taste and Smell

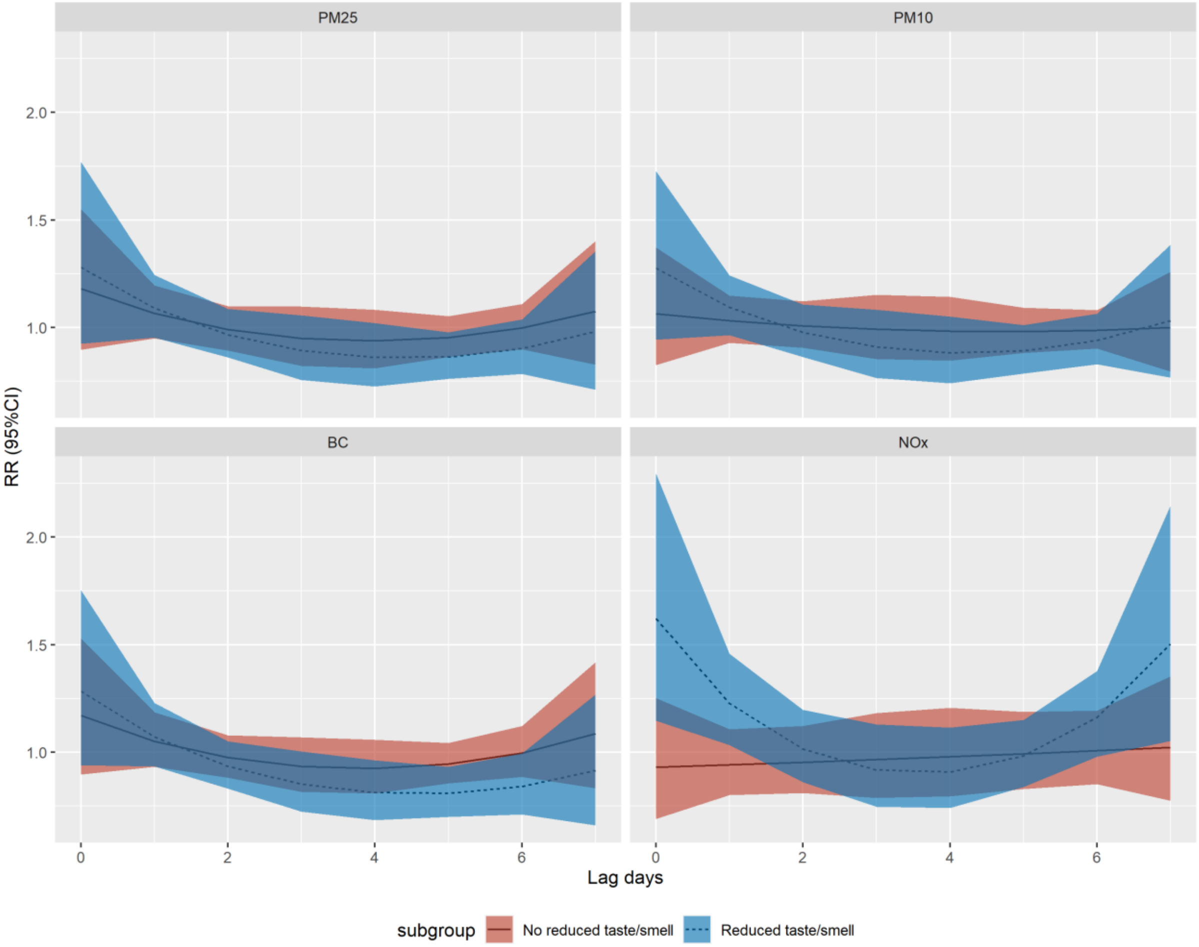

© 2022 Yu Z et al. *JAMA Network Open*.

eFigure 12. Lag-Specific Relative Risks for SARS-CoV-2 Infection Associated With per-IQR Increase in Short-term Air Pollution Exposure Stratified by Self-reported Nasal Congestion

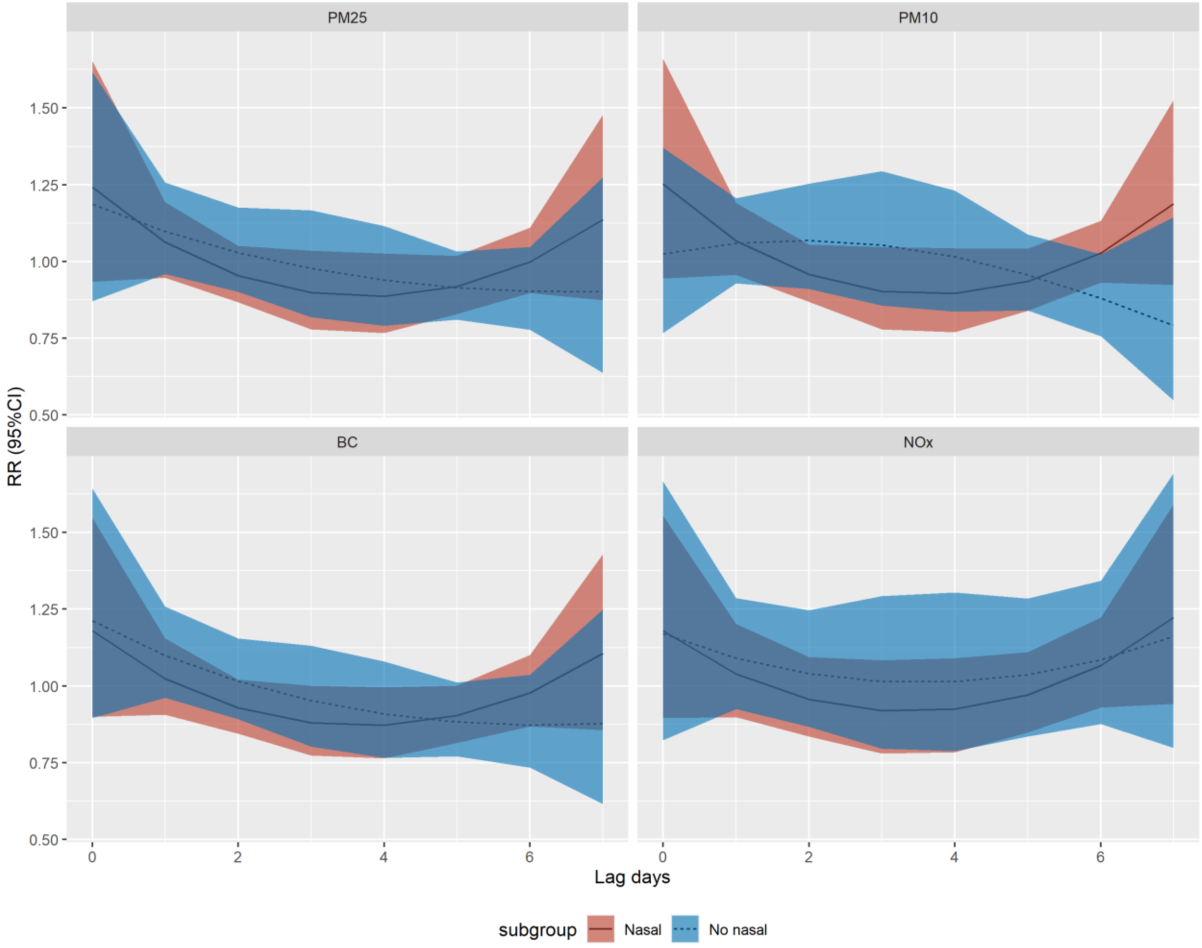

© 2022 Yu Z et al. *JAMA Network Open*.

eFigure 13. Lag-Specific Relative Risks for SARS-CoV-2 Infection Associated With per-IQR Increase in Short-term Air Pollution Exposure Stratified by Self-reported Sniffle

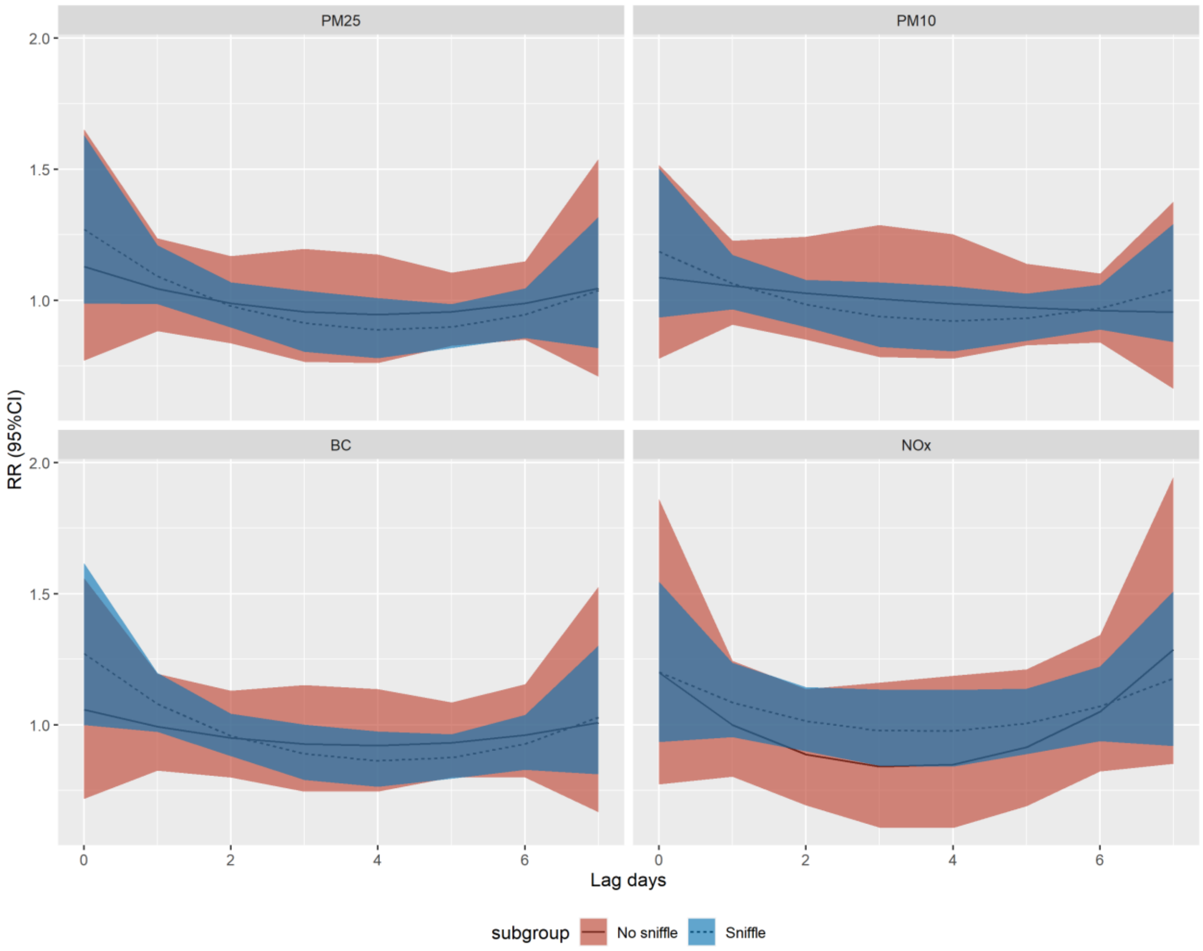

© 2022 Yu Z et al. *JAMA Network Open*.

eFigure 14. Lag-Specific Relative Risks for SARS-CoV-2 Infection Associated With per-IQR Increase in Short-term Air Pollution Exposure Stratified by Self-reported Breathing Difficulty

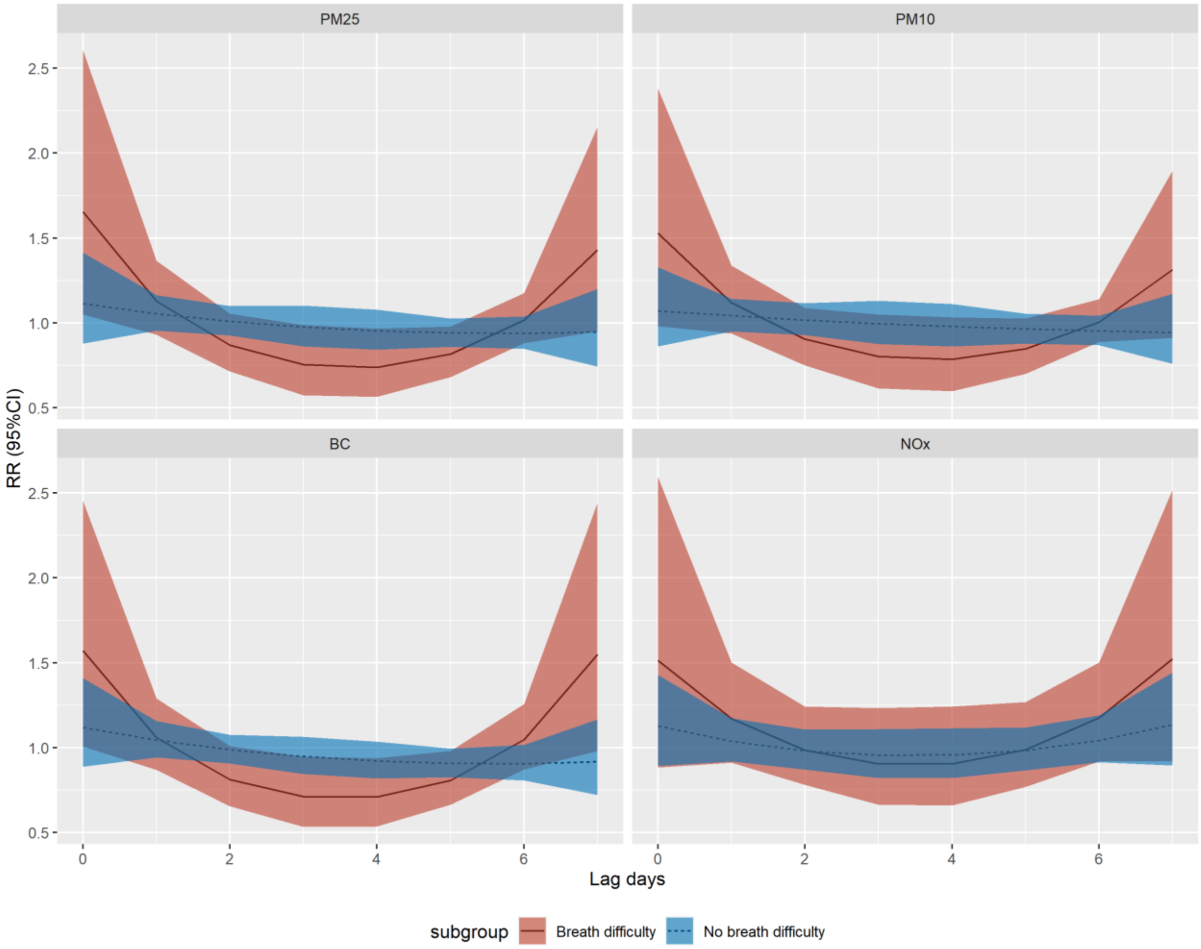

© 2022 Yu Z et al. *JAMA Network Open*.
