# Supplementary material for: Association of Short-term Air Pollution Exposure With SARS-CoV-2 Infection Among Young Adults in Sweden
Source: JAMA Netw Open. 2022 Apr 20;5(4):e228109. doi: 10.1001/jamanetworkopen.2022.8109 (PMC9021914; doi:10.1001/jamanetworkopen.2022.8109)
Supplement: Supplement 2. — Nonauthor Collaborators [file jamanetwopen-e228109-s002.pdf]

Supplemental Online Content: Nonauthor Collaborators

*Indicates required information. Only first name, last name, and suffix will appear in PubMed.

***Group Name(s): BAMSE COVID-19 Study Group**

***First Name and Middle Initial(s)**	***Last Name**	***Suffix (eg, Jr, III)**	Academic Degrees	Institution	Location (city, state/province, country)	Role or Contribution, eg, chair, principal investigator	Group (if more than 1 Group listed in the byline) and/or Subgroup (eg, Steering Committee)	
Catarina	Almqvist		Prof.	Department of Medical Epidemiology and Biosta	Stockholm, Sweden	BAMSE Steering group		
Niklas	Andersson		MSc	Unit of Environmental Epidemiology, Institute of Environmental Medicine, Karolinska Institute	Stockholm, Sweden	BAMSE secretariat		
Natalia	Ballardini		MD,PhD	Department of Clinical Science and Education Sö	Stockholm, Sweden	BAMSE Steering group		
Anna	Bergström		Assoc. Prof	Unit of Environmental Epidemiology, Institute of Environmental Medicine, Karolinska Institute	Stockholm, Sweden	BAMSE Steering group, PI		
Sophia	Björkander		PhD	Department of Clinical Science and Education Sö	Stockholm, Sweden	Immunology /Research and Cohort material collection		
Petter	Brodin		Prof.	SciLifeLab; Department of Women's and Childre	Stockholm, Sweden	Pediatric imunology		
Anna	Castel			Department of Clinical Sciences and Education, S	Stockholm, Sweden	Responsible research nurse		
Sandra	Ekström		PhD	Centre for Occupational and Environmental Med	Stockholm, Sweden	BAMSE secretariat		
Antonios	Georgelis		Assoc. Prof	Centre for Occupational and Environmental Med	Stockholm, Sweden	BAMSE Steering group, PI		
Lennart	Hammarström		Prof.	Department of Laboratory Medicine, Klin Immunologi o Transfusionsmedicin, Karolinska Institutet	Stockholm, Sweden	Immunology		
Qiang	Pan-Hammarström		Prof.	Department of Biosciences and Nutrition, Karolinska Institutet	Stockholm, Sweden	B cell and immunoglobulin gene diversification		
Jenny	Hallberg		PhD	Department of Clinical Sciences and Education, S	Stockholm, Sweden	Lung function		
Christer	Jansson		Prof.	Department of Medical Sciences: Respiratory, Al	Uppsala, Sweden	BAMSE Steering group		

Supplemental Online Content: Nonauthor Collaborators

*Indicates required information. Only first name, last name, and suffix will appear in PubMed.

***First Name and Middle Initial(s)**	***Last Name**	***Suffix (eg, Jr, III)**	Academic Degrees	Institution	Location (city, state/province, country)	Role or Contribution, eg, chair, principal investigator	Group (if more than 1 Group listed in the byline) and/or Subgroup (eg, Steering Committee)	
Maura	Kere			Department of Clinical Sciences and Education, S	Stockholm, Sweden	Epidemiolog y		
Inger	Kull		Prof.	Department of Clinical Science and Education Sö	Stockholm, Sweden	BAMSE Steering group, PI		
André	Lauber			Centre for Occupational and Environmental Med	Stockholm, Sweden	BAMSE secretariat		
Alexandra	Lövquist		PhD	Centre for Occupational and Environmental Med	Stockholm, Sweden	BAMSE secretariat		
Erik	Melén		Prof.	Department of Clinical Science and Education Sö	Stockholm, Sweden	BAMSE Steering group, PI		
Jenny	Mjösberg		Assoc. Prof	Department of Medicine, Huddinge, Centrum för	Stockholm, Sweden	Innate immunocells		
Ida	Mogensen		PhD	Karolinska Institutet, Institutionen för kliniska ve	Stockholm, Sweden	Lung function and inflammatio n in asthma		
Lena	Palmberg		Prof.	Integrative Toxicology, Institute of Environmenta	Stockholm, Sweden	BAMSE Steering group		
Göran	Pershagen		Prof.	Unit of Environmental Epidemiology, Institute of Environmental Medicine, Karolinska Institute	Stockholm, Sweden	BAMSE Steering group		
Niclas	Roxhed		Assoc. Prof	KTH Royal Institute of Technology; MedTechLabs	Stockholm, Sweden	Sampling technology		
Jochen	Schwenk		Prof.	Science for Life Laboratory, Division of Affinity Pr	Stockholm, Sweden	Proteomics		
